# *In-vitro* transcriptomic profiling of indigenous Gaddi vis-à-vis exotic Labrador dogs: insights from systems biology

**DOI:** 10.3389/fvets.2025.1489905

**Published:** 2025-06-20

**Authors:** Jaswinder Kaur, Manu Mohan, Bilawal Singh, Ram Saran Sethi, Deepti Narang, Simarjeet Kaur, Chandra Sekhar Mukhopadhyay

**Affiliations:** ^1^Department of Bioinformatics, College of Animal Biotechnology, Guru Angad Dev Veterinary and Animal Science University, Ludhiana, India; ^2^Department of Microbial and Environmental Biotechnology College of Animal Biotechnology, Guru Angad Dev Veterinary and Animal Science University, Ludhiana, India; ^3^Department of Veterinary Gynecology and Obstetrics, Guru Angad Dev Veterinary and Animal Science University, Ludhiana, India; ^4^Dean College of Animal Biotechnology, Guru Angad Dev Veterinary and Animal Sciences University, Ludhiana, Punjab, India; ^5^Department of Veterinary Microbiology, College of Veterinary Sciences, Guru Angad Dev Veterinary and Animal Science University, Ludhiana, India; ^6^Department of Animal Genetics and Breeding, College of Veterinary Sciences, Guru Angad Dev Veterinary and Animal Science University, Ludhiana, India

**Keywords:** Gaddi dogs, Labrador dog, transcriptome, miRNAome, immune responses, TLR-ligands

## Abstract

**Introduction:**

The domestication of dogs is regarded as an evolutionary adaptation influenced by artificial selective pressures, leading to the fruition of diverse canine breeds across regions. Indigenous breeds, developed in tandem with local environments, display unique conformations and disease resistance, yet many remain understudied at the molecular level. The Gaddi dog, originating in the northern parts of India and used by local tribes for livestock guarding, exemplifies such a breed with potential for transcriptomic research. Despite its vital role, it remains unrecognized by the National Bureau of Animal Genetic Resources (NBAGR). This study addresses the gaps in understanding the genetics and immune responses of Indigenous breeds, emphasizing their importance as holders of unique genetic heritage. This study explores the molecular profiles of Indigenous Gaddi dogs and exotic Labrador retrievers, focusing on their immune responses to TLR ligand-induced infections.

**Methods:**

The mRNA and miRNA sequencing were performed separately using the Illumina NovaSeq 6,000 platform (150 bp). The study involved comparing the Control group (i.e., without treatment of any TLR-ligand) with each of the Poly I: C, LPS, and CpG ODN-treated groups for Labrador and Gaddi dogs. Functional enrichment analysis of differentially expressed genes (DEGs) (fold change >3 and <−3, *p* < 0.05) was conducted to identify enriched pathways in each breed.

**Results:**

The analysis revealed that Labrador dogs had more DEGs across all treatment groups than Gaddi dogs. The enriched pathways in Labradors included Th1, Th2, Th17 cell differentiation, and T-cell receptor signaling. In contrast, Gaddi dogs significantly enriched ‘Wnt’ signaling, T cell activation, and immune regulation pathways. The differential expression (DE) analysis of miRNA-Seq results indicated that Labradors had more DE miRNAs (with expression levels of the original level >1.5 and <−1.5), such as miR-204, miR-206, miR-106a, miR-132, miR-335, and miR-676, which help regulate inflammation, autophagy, and immune responses. Gaddi dogs had unique miRNAs (miR-551 and miR-1249) associated with tumor suppression and inflammation.

**Discussion:**

The study highlights distinct immunological profiles between Labrador and Gaddi dogs, with no shared genes responding to TLR-ligand stimulation. The functional enrichment of miRNA targets demonstrated consistent regulatory patterns at both the mRNA and miRNA levels. These findings emphasize the importance of preserving the genetic diversity of indigenous Gaddi dogs and utilizing advanced sequencing techniques to explore immunological diversity for disease resistance and the selection of breeding individuals.

## 1 Introduction

Domestication of dogs is primarily viewed as an evolutionary response to a new, human-dominated environment, influenced by artificial selective pressures exerted by humans (1). India harbors a diversified and well-integrated fauna of all Indigenous animals, including different breeds/strains/genetic groups of dogs, such as Mudhol Hound, Chippiparai, Kanni, etc. Native animals are those species that have their origin within a particular locality and, over time, developed in tandem with the prevailing climatic and environmental conditions of the Locality (69). These native dogs vary in conformation, exhibiting differential genetic predisposition to diseases and variable responses to infections (2). It is worth noting that molecular characterization of many of these genetic groups is still warranted. The Gaddi dog is an important breed and has recently been recognized by the National Bureau of Animal Genetic Resources (NBAGR)^1^ (Accession number INDIA_DOG_0600_GADDI_19004). The Gaddi dogs or “Shepherds’ dogs” are one such breed originating from the northern parts of India, majorly Himachal Pradesh, Uttarakhand, Jammu, and Kashmir, and some parts of Punjab, used by the Gaddi tribe for guarding livestock in hilly terrains. Moreover, Gaddi dogs are ideal for transcriptomic studies since these populations of free-ranging dogs are pre-adapted to harsh climatic conditions and high altitudes. These Gaddi dogs, being “Free-ranging dogs” (FRDs), showed signs of diversifying selection in genes whose function is in reproduction, immunity, and chemosensory perception, suggesting adaptations for independent survival (3). Such adaptive characteristics may benefit dogs in human-altered landscapes through hybridization and gain enhanced immunity against environmental strains and diseases (4).

Blood transcriptomic profiling is a valuable tool for evaluating host immune responses to specific stimulants or pathogens (5). Peripheral blood mononuclear cells (PBMCs) are vital players in the immune system, crucial for defending against infections (6). PBMCs have been frequently employed as an *ex vivo* model for studying cytokine gene expression following stimulation with purified toll-like receptor (TLR) ligands or live probiotics (7, 8). MicroRNAs (miRNAs), small non-coding RNAs (~19–23 nucleotides), are essential regulators of protein expression and have significant roles in development, physiological processes, and disease. Their ability to interact dynamically with multiple target genes and rapidly alter expression in response to external stimuli underscores their importance in maintaining cellular homeostasis (9–11). Moreover, research reveals that miRNA expression is adjusted under stressful conditions to manage the stress response (12). In our lab, we conducted similar research investigating differentially expressed miRNAs in buffalo PBMCs exposed to various TLR ligands (13, 14, 77). RNA sequencing (RNA-seq) provides a high-throughput approach for comprehensive transcriptome analysis, offering detailed insights into gene expression (15, 72). Understanding miRNA-mRNA regulatory networks is critical for elucidating gene expression at the post-transcriptional level. However, in silico models for predicting miRNA-mRNA interactions often have limited accuracy and context-specific relevance. A more robust strategy involves integrating actual mRNA and miRNA transcriptomic and miRNAomic data with computational predictions (16, 17).

The present study was designed to explore the prominent differences in the transcriptome and miRNAome profiles of Indigenous Gaddi dogs and exotic Labrador retrievers because of their distinct origin and genetic background. This study was undertaken to elucidate the molecular profile of PBMCs under *in vitro* simulated viral and bacterial infections, using Toll-like receptor (TLR) ligands—TLR4, TLR3, and TLR9. First, literature on mRNA and miRNA responsive expression to these conditions in dogs is limited. Our study has addressed questions concerning breed-specific immune responses and the genetics involved in their adaptation, underscoring preservation and research on Indigenous dog breeds as holders of unique genetic heritage.

## 2 Materials and methods

### 2.1 Ethical permissions

**Figure 1:**
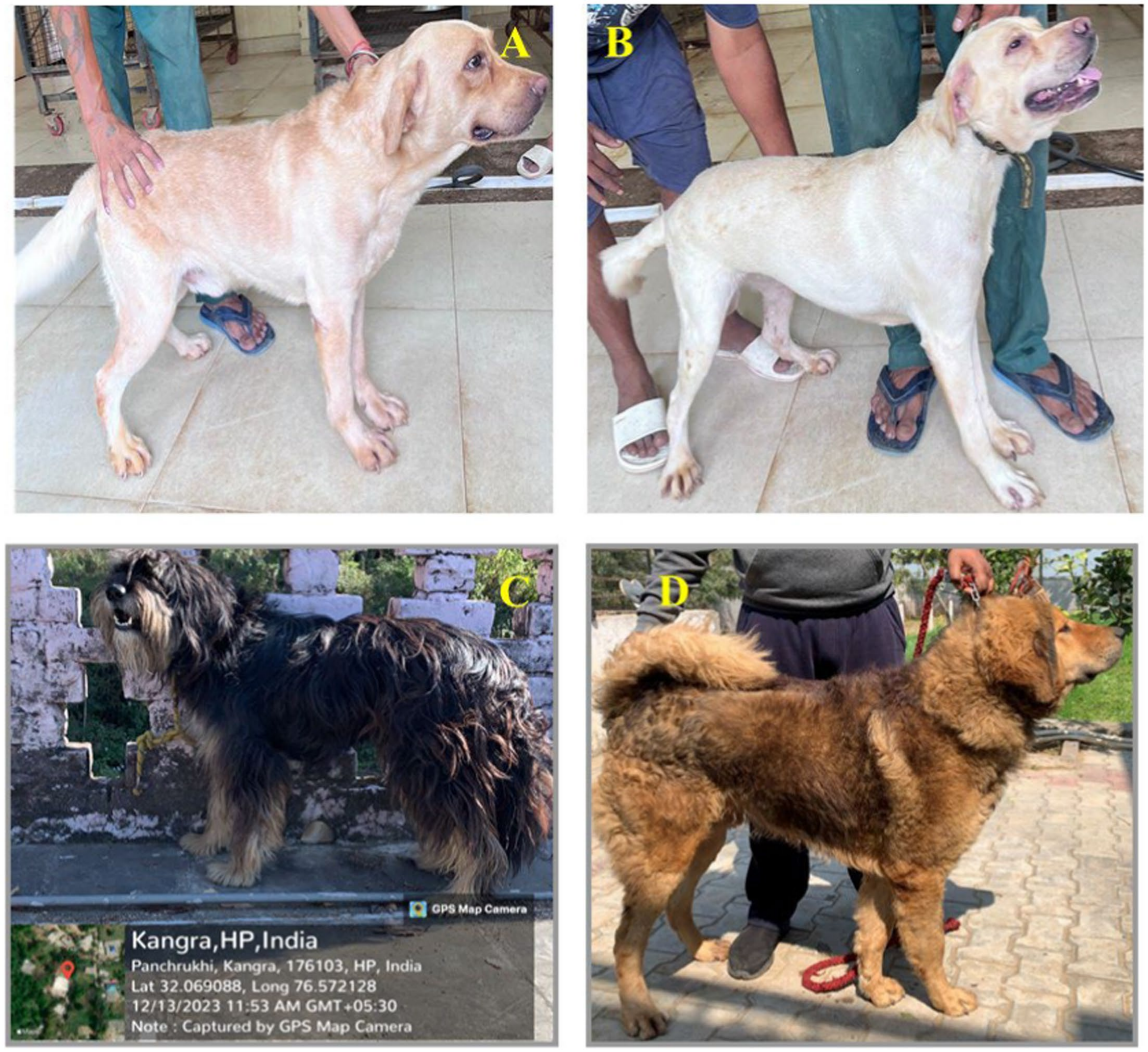
Collection of experimental blood samples from the exotic Labrador breed and indigenous *Gaddi* dogs: **(A)** Labrador dog number 3, **(B)** Labrador dog number 6, **(C)** Baan *Gaddi* dog from Palampur, H.P, **(D)** *Gaddi* dog from Kennel, Barnala, Punjab.

Blood sample collection from Labrador dogs (Exotic) and Gaddi dogs (Indigenous) was approved by the Institutional Animal Ethics Committee (IAEC) (GADVASU/2022/IAEC/64/11) on 11th March 2022. Labrador Retriever samples were collected from the Dog House at Guru Angad Dev Veterinary and Animal Sciences University, Ludhiana, India, while Gaddi dog samples were collected from Palampur, Himachal Pradesh (Supplementary Table 1; Figure 1). Gaddi dog blood samples were processed for PBMC extraction and *in vitro* treatment at the Department of Veterinary Microbiology, Dr. G.C. Negi College of Veterinary and Animal Sciences, Chaudhary Sarwan Kumar Himachal Pradesh Krishi Vishvidyalaya, Palampur. The study was conducted in duplicates with two biological replicates of an adult male Gaddi dog and an adult male Labrador Retriever of a similar age group. Experimental animals were selected from 5 to 6 randomly chosen male dogs of each genetic group during winter, based on their health status determined by TLC and DLC tests. Peripheral blood (3–5 ml) was aseptically collected from the cephalic vein using 500 μl of 0.5 M EDTA as an anticoagulant. Immediately after collection, TLC and DLC tests were performed to confirm the health status of the dogs (Supplementary Table 2). Blood samples were processed within 2 h. PBMCs were isolated using Hisep™ LSM (Himedia) through density gradient centrifugation at 500 × *g* for 30 min, then carefully removing the buffy coat. Cells were washed with 1X phosphate-buffered saline (pH 7.4) at 500 × g for 10 min, and viability was assessed using the trypan blue exclusion method. Approximately 2 × 10^6 PBMCs were counted using a hemocytometer and cultured in 6-well plates in RPMI-1640 (HiMedia) supplemented with 10% FBS (HiMedia) and 1% penicillin–streptomycin (Thermo Fisher Scientific) at 37°C in a 5% CO₂ incubator.

For mRNA and miRNA sequencing, PBMCs were divided into three experimental groups—TLR3 ligand (Poly I: C, InvivoGen), TLR4 ligand (Lipopolysaccharide, ChemCruz), and TLR9 ligand (CpG ODN, Invitrogen)—along with a control group for both Labrador and Gaddi dogs in duplicates. PBMCs were challenged with Poly I: C (50 μg/ml) for 12 h, LPS (100 ng/ml) for 6 h, and CpG ODN (10 μg/ml) for 12 h (13) (Table 1). All protocols were conducted under sterile conditions to prevent contamination, and replicate samples were processed identically.

**Table 1:** Experimentation on different groups of cultures by challenging the PBMCs with TLR ligands for mRNA Seq.

S. No.	Treatment	Control
1	TLR 3 Ligand, (Poly I: C) @ 50 μg/ml for 12 h	No treatment of Poly I: C
2	TLR 4 Ligand, (LPS) @ 100 ng/ml for 6 h	No treatment of LPS
3	TLR 9 Ligand, (CpG ODN) @ 10 μg/ml for 12 h	No treatment of CpG-ODN

### 2.2 RNA isolation and library construction

#### 2.2.1 mRNA isolation and library preparation

The cultured PBMCs were subjected to mRNA and miRNA extraction. For mRNA extraction, the sequencing agency used the TRIzol method. The quantity of RNA was determined using the Qubit® 4.0 fluorometer (Thermos Fisher Scientific), and its quality was assessed by examining a 1% agarose gel. The paired-end sequencing library was prepared using the KAPA mRNA HyperPrep Kit for Illumina (KAPA Biosystems). The amplified libraries were assessed at Unipath Specialty Laboratory Ltd. on the TapeStation 4,150 (Agilent Technologies) using HSD100 ScreenTape®.

#### 2.2.2 miRNA isolation and library preparation

miRNA extraction was done using the Direct-zol™ RNA MicroPrep (Zymo Research) kit. The Qubit™ RNA BR Assay Kits (Invitrogen, ThermoFisher Scientific Inc.) and Qubit™ 2.0 Fluorometer (Life Technologies, Thermo Fisher Scientific Inc.) were used (by) to quantify the small RNA extracted from the PBMCs. The QIAseq (QIAGEN) miRNA NGS kit was used for miRNA library preparation, including efficient 3' and 5' adapter ligation and cDNA synthesis with a Unique Molecular Identifier (UMI), followed by library amplification.

### 2.3 RNA sequencing and bioinformatics analysis

#### 2.3.1 mRNA sequencing

Biological replicates from each simulated group and a control group of Labrador and Gaddi dogs were pooled into single samples for NGS. mRNA extraction Sequencing and initial Bioinformatic analysis were performed by Unipath Specialty Laboratory Ltd. in Ahmedabad, Gujarat, using the Illumina NovaSeq 6,000 platform to generate 2 × 150 bp paired-end reads. The raw sequencing data underwent preprocessing, including adapter removal and filtering out low-quality bases using Trimgalore (version 0.6.4) (18). The reads were aligned to a reference genome using the STAR aligner (version 2.7.10a) (19). Transcript assembly was then conducted using StringTie (version 2.2.1) (20, 21). The abundance of the transcripts in all samples was estimated using StringTie. A Python program (prepDE.py) was utilized to extract the read count information directly from the files generated by StringTie. Differential gene expression was inferred between samples by applying the R package edgeR (22), a Bioconductor package based on the negative binomial distribution method. A comprehensive DEG analysis for functional enrichment was conducted, focusing on DEGs with logFC ≥ 3 or ≤−3 and a *p*-value <0.05. GO analysis of the DEGs was performed using the DAVID database^2^ to explore their functions and associated biological processes. To identify the signaling pathways linked to these DEGs, analyses were performed using KEGG,^3^ Reactome,^4^ and PANTHER^5^ (23). The STRING database^6^ was used to construct a protein–protein interaction (PPI) network for the DEGs (24).

#### 2.3.2 miRNA sequencing

miRNA libraries for each simulated group of Labrador and Gaddi dogs were pooled into single samples for Illumina NovaSeq 6,000 NGS, and initial bioinformatics analysis was done by Life Cell Diagnostics Ltd., ChennaiLife Cell Diagnostics Ltd., and Chennai did miRNA extraction, sequencing, and initial bioinformatics analysis. The preprocessing steps encompassed trimming adaptor sequences and filtering out too-small reads using Trimgalore v0.6.7 (25) and Cutadapt v1.18 (26). Reads were aligned to the Dog reference genome (GCF_014441545.1) using miRDeep2’s mapper.pl., script and miRNA identification were performed with known and novel miRNA parameters. Mature miRNAs were referenced from miRBase v22.1 for prediction (27, 79). Differential expression analysis was performed using DESeq2 v1.34.0, with read counts normalized and analyzed for differential expression (28). miRNAs with an absolute log2 fold change ≥ 1 and a *p*-value ≤ 0.05. threshold false discovery rate <0.05 and fold change >1.5. Potential target genes of differentially expressed miRNAs were predicted using three online tools: TargetScan v6.2, MiRTarBase, and miRDB (29–31). Target transcripts were screened based on their functions to identify those involved in innate immunity and disease pathogenesis. Gene ontology classification of miRNA targets into biological processes and molecular functions was performed using the PANTHER Classification System (81). The STRING database Further analyses were conducted using the DAVID database KEGG pathways, Reactome. The STRING database (24) was employed to construct a protein–protein interaction (PPI) network for the DEGs. Graphical data representations were created using SRPLOTS^7^ (32).

### 2.4 qRT-PCR validation of differentially expressed (DE) mRNAs and miRNAs

The top 40 dysregulated DE genes and DE miRNA from both the dog breeds were identified across three treatment groups (Control vs. LPS, Control vs. Poly I: C, and Control vs. CpG ODN). This was followed by selecting commonly dysregulated genes across the treatment groups and further screening for functional enrichment using Gene Ontology (GO) terms, highlighting disease-associated genes. Four key mRNA-Seq DE genes were identified: PPP1R12A (downregulated) and SLMAP (upregulated) in Labrador, and WSB2 (upregulated) and ATP6AP2 (downregulated) in Gaddi dogs (Supplementary Table 3). For miRNA targets, seven common genes were identified (Supplementary Tables 4, 5). ARMC8 (upregulated in all the treatment groups), FIBCD1(Control vsSTARD13 (Control vs. Poly I: C), VPS13D (Control vs. LPS), and STARD13 (Control vs. CpG) were downregulated in Labrador dog PBMC. On the other hand, SLC24A2 (common in Control vs. LPS & Poly I: C), KATNBL1 (Control vs. CpG) were up-regulated, and STARDB (downregulated) in all the groups of *Gaddi* dogs PBMCs. This comprehensive selection of genes and target predictions was subsequently used for qPCR validation. These 11 genes were selected for qPCR validation, including mRNA Seq and miRNA Seq. Primers were designed using NCBI Primer-BLAST and Primer3 Plus, and samples were analyzed in triplicate with melt curve analyses to ensure PCR product specificity. Total RNA was extracted from each Labrador dog (dog no. 1 and 2) and Gaddi dogs (Dog no. 1 and 2) PBMCs using the TRIzol method, and cDNA was synthesized with the Takara PrimeScript II Kit. RT-PCR validation was performed using the Bio-Rad CFX96 system and the Qiagen miScript SYBR Green PCR Kit. Relative gene expression was normalized to GAPDH and ACTB and determined using the ΔΔCt method (33).

## 3 Results

### 3.1 mRNA sequencing and data summary

To begin exploring the systems biology of PBMCs of Labrador and Gaddi dog transcriptome upon various TLR ligand treatments (Poly I: C, LPS, and CpG ODN), we performed an RNA-seq experiment. Sixteen libraries were prepared (8 for Labrador dogs) and (8 for Gaddi dogs) for three treatments and a control in duplicates. Then, it sequenced upon pooling the duplicate respective treatment within the breed. The raw mRNA Seq and miRNA Seq data were uploaded under the project accession number SRA ID PRJNA1035251. The data output and the mapped reading statistics for Labrador and Gaddi dogs’ control and treatment samples are shown in Tables 2 and 3, respectively.

**Table 2:** mRNA sequencing data statistics for Labrador and *Gaddi* dog PBMCs across the treatment groups.

Samples	Total reads in R1	Total reads in R2	Total reads (R1 + R2)	Total bases (R1 + R2)	Total data (GB)
Lab-Ctrl	14,369,165	14,369,165	28,738,330	4,569,394,470	4.57
Lab-PolyIC	12,163,001	12,163,001	24,326,002	4,306,459,668	3.87
Lab-LPS	13,621,987	13,621,987	27,243,974	4,331,791,866	4.33
Lab-CpG	13,542,326	13,542,326	27,084,652	4,306,459,668	4.31
*Gaddi*—Ctrl	3,81,34,023	3,81,34,023	7,62,68,046	12,126,619,314	12.13
*Gaddi*—PolyIC	3,76,60,926	3,76,60,926	7,53,21,852	11,976,174,468	11.98
*Gaddi*—LPS	1,44,70,705	1,44,70,705	2,89,41,410	4,601,684,190	4.6
*Gaddi*—CpG	2,88,23,230	2,88,23,230	5,76,46,460	9,165,787,140	9.17

This colour gradient helps to highlight patterns or contrasts in the dataset and emphasizes biologically or statistically significant trends in data set.

**Table 3:** Reference genome mapping statistics of RNA-Seq reads in Labrador and *Gaddi* dog PBMCs across the treatment groups.

Sample Name	Total reads	No. of mapped reads	% of mapped read	Uniquely mapped reads	% uniquely mapped reads
Lab-Ctrl	14,360,062	12,147,888	84.59	11,654,493	81.16
Lab-Poly IC	12,149,173	11,587,391	95.38	10,925,233	89.93
Lab-LPS	13,611,015	12,837,078	94.31	12,169,477	89.41
Lab-CpG	13,530,667	12,769,944	94.38	11,950,684	88.32
*Gaddi*-Ctrl	38,004,280	34,125,094	89.79	33,005,358	86.85
*Gaddi*-Poly IC	28,688,937	25,425,654	88.63	24,650,104	85.92
*Gaddi*-LPS	37,301,612	31,347,529	84.04	29,540,540	79.19
*Gaddi*-CpG	14,448,463	13,565,486	93.89	13,213,308	91.45

This colour gradient helps to highlight patterns or contrasts in the dataset and emphasizes biologically or statistically significant trends in data set.

#### 3.1.1 Identification of differentially expressed genes

Differential gene expression was inferred between samples using the R package edgeR (22). A comprehensive DEG analysis for functional enrichment was conducted, focusing on the DEGs with a log of fold change (logFC) ≥ 3 or ≤ − 3 and a *p*-value < 0.05. The differential expression analysis revealed varying numbers of dysregulated transcripts across the experimental groups in Labrador and Gaddi dogs, as shown in Table 4.

**Table 4:** Significant differentially expressed genes (DEGs) in Labrador and Gaddi dogs (logFC ≥ 3 or ≤ − 3, *p*-value < 0.05).

S. No.	Exp-group	Total-dysregulated-transcripts-count	DEG-LFC>|3|
1	Ctrl-PolyIC-Lab	5,476	555
2	Ctrl-LPS-Lab	5,622	694
3	Ctrl-CpG-Lab	4,979	549
4	Ctrl-PolyIC-*Gaddi*	1,483	204
5	Ctrl-LPS-*Gaddi*	3,646	334
6	Ctrl-CpG-*Gaddi*	2,417	1,072

This colour gradient helps to highlight patterns or contrasts in the dataset and emphasizes biologically or statistically significant trends in data set.

**Figure 2:**
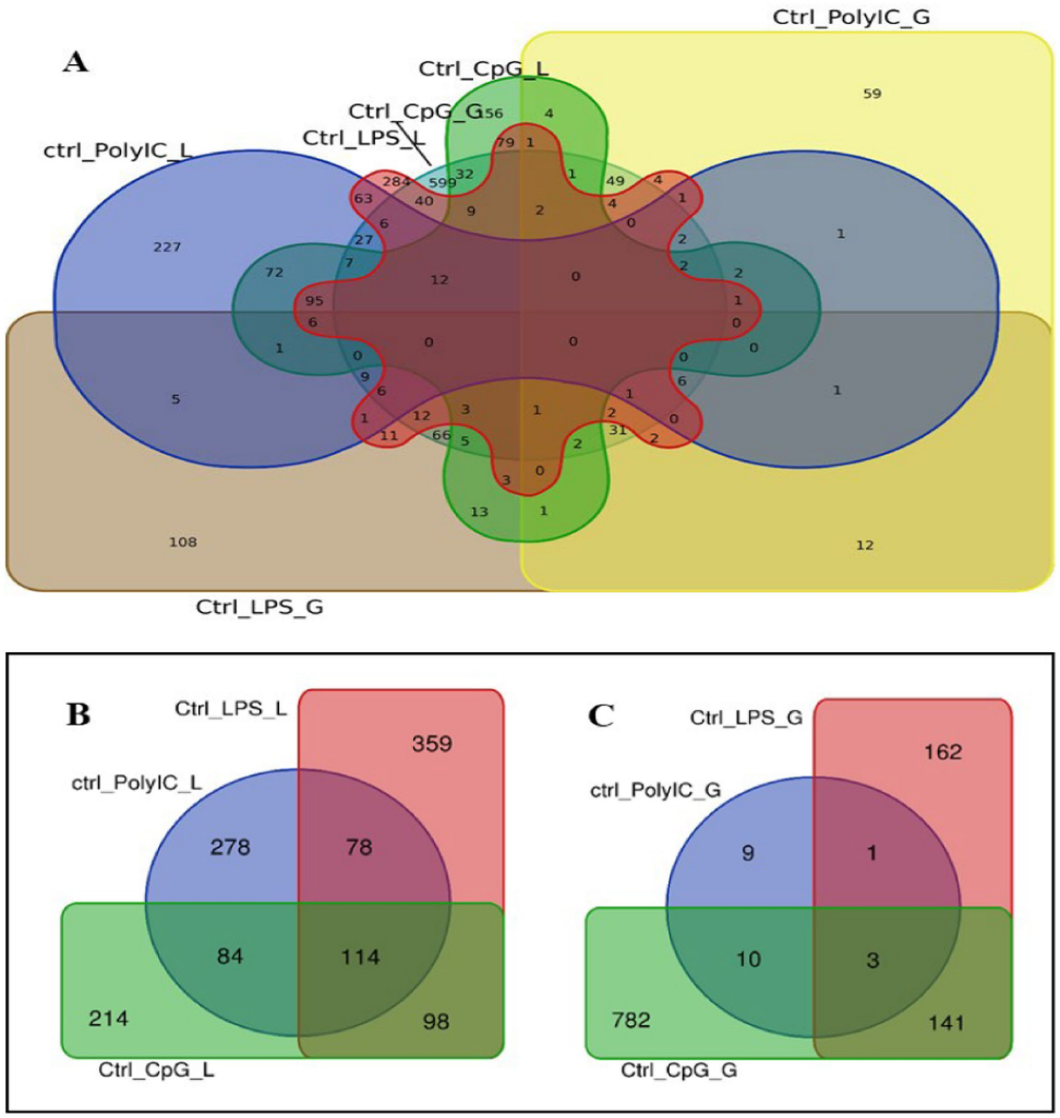
Venn diagram analysis of DEGs in Labrador and *Gaddi* dogs with logFC ≥ 3 or ≤−3 and *p*-value < 0.05. **(A)** Comparison between the Labrador DEGs (Ctrl-Poly IC, Ctrl-LPS, and Ctrl-CpG) and *Gaddi* dog DEGs (Ctrl-Poly IC, Ctrl-LPS, and Ctrl-CpG). **(B)** Venn diagram of Labrador experimental groups. **(C)** Venn diagram of *Gaddi* dog experimental groups.

**Figure 3:**
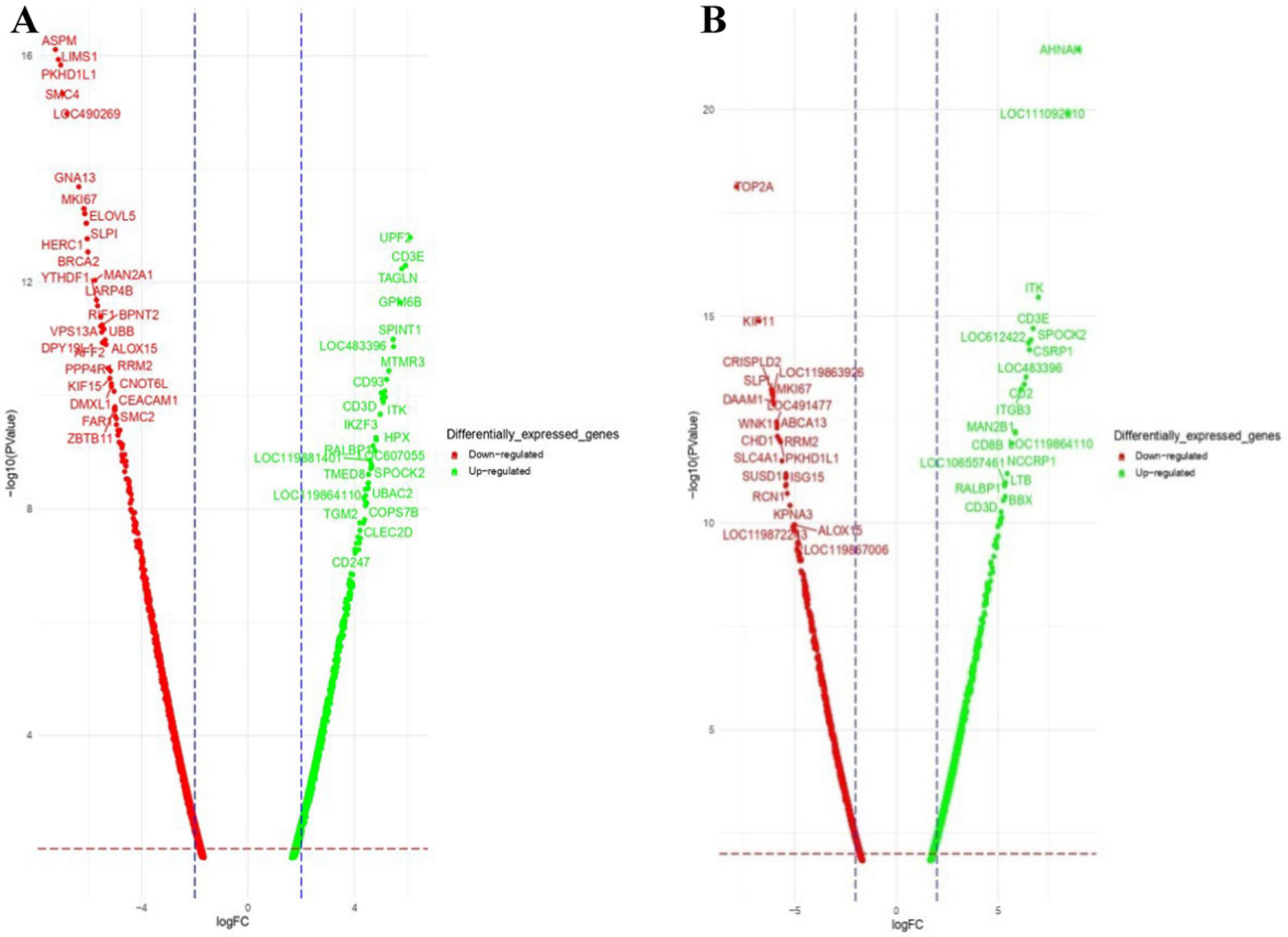
Volcano plot of dysregulated DEGs in Labrador dogs **(A)** Ctrl-Poly IC and **(B)** Ctrl-LPS. The volcano plots display the log2 fold change (logFC) and -log10 *p*-value of differentially expressed genes (DEGs) with logFC ≥3 (highlighted green) or ≤−3 (highlighted red) and *p*-value < 0.05.

**Figure 4:**
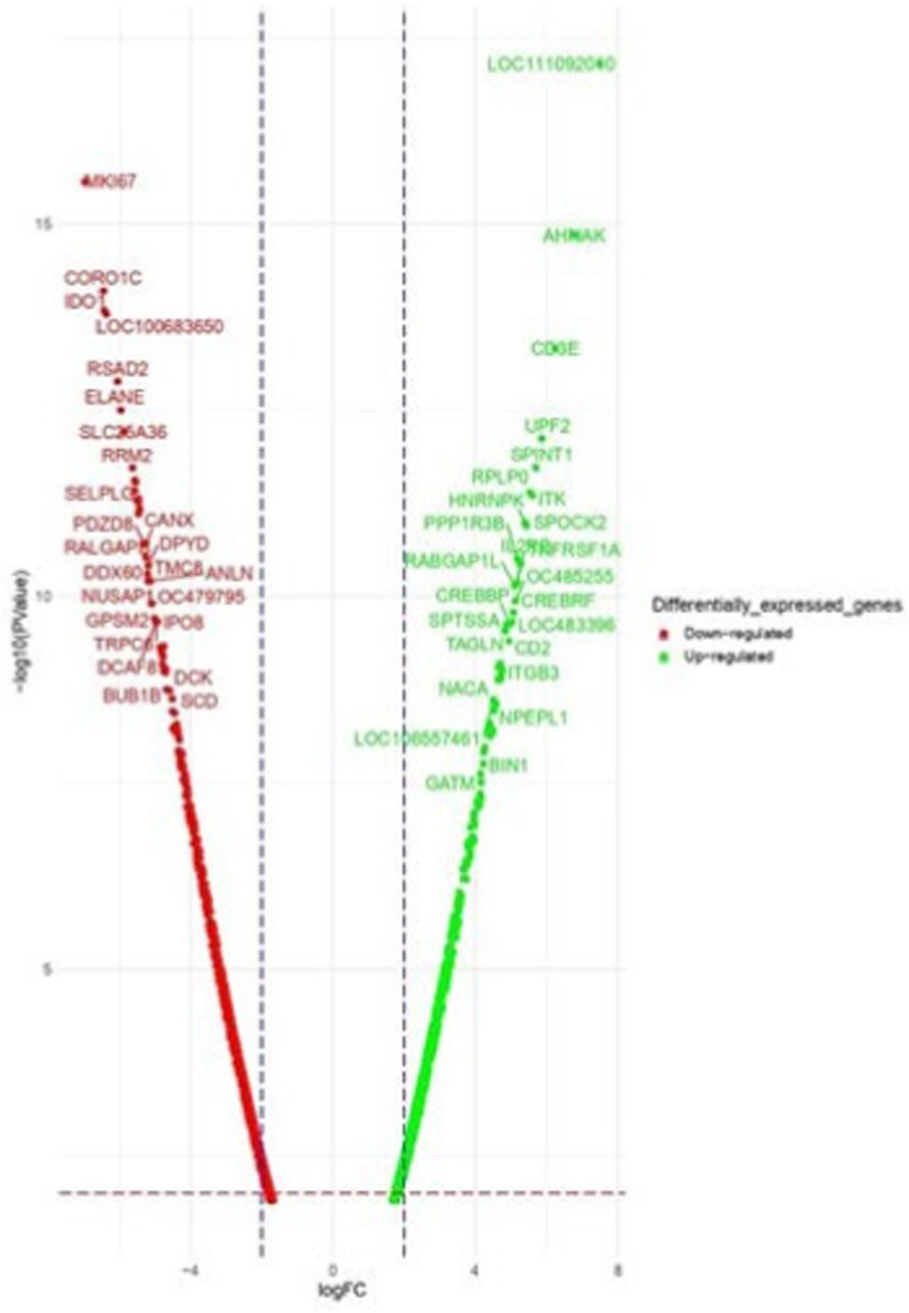
Volcano plot of dysregulated DEGs in Labrador dogs. Ctrl-CpG. The volcano plot displays the log2 fold change (logFC) and -log10 *p*-value of differentially expressed genes (DEGs) with logFC ≥ 3 (highlighted green) or ≤−3 (highlighted red) and *p*-value < 0.05.

**Figure 5:**
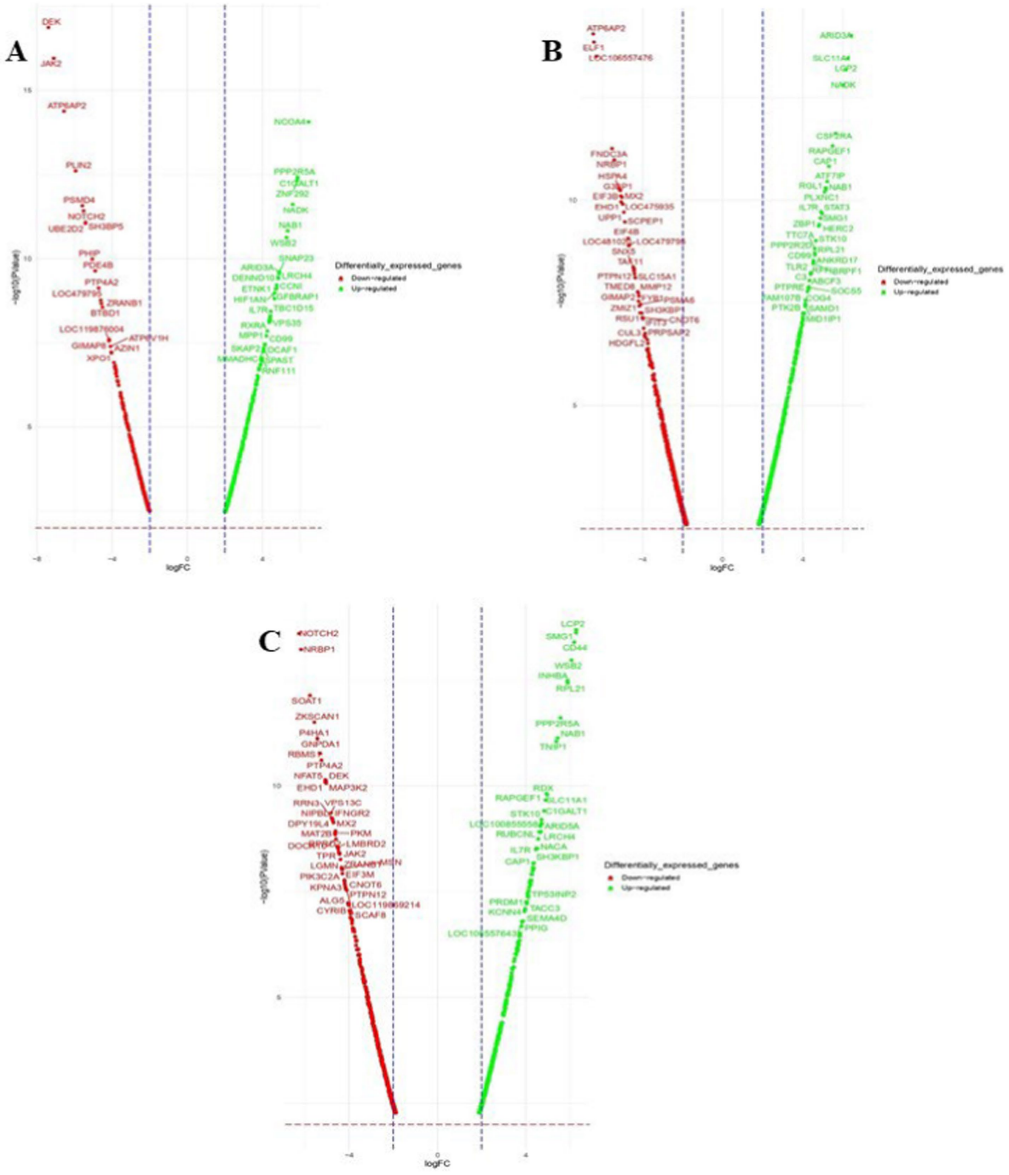
Volcano plots of dysregulated DEGs in *Gaddi* dogs. (A) Ctrl-Poly IC and B. Ctrl-LPS, C. Ctrl-CpG. The volcano plots display the log2 fold change (logFC) and -log10 *p*-value of differentially expressed genes (DEGs) with logFC ≥ 3 (highlighted green) or ≤ − 3 (highlighted red) and *p*-value < 0.05.

**Figure 6:**
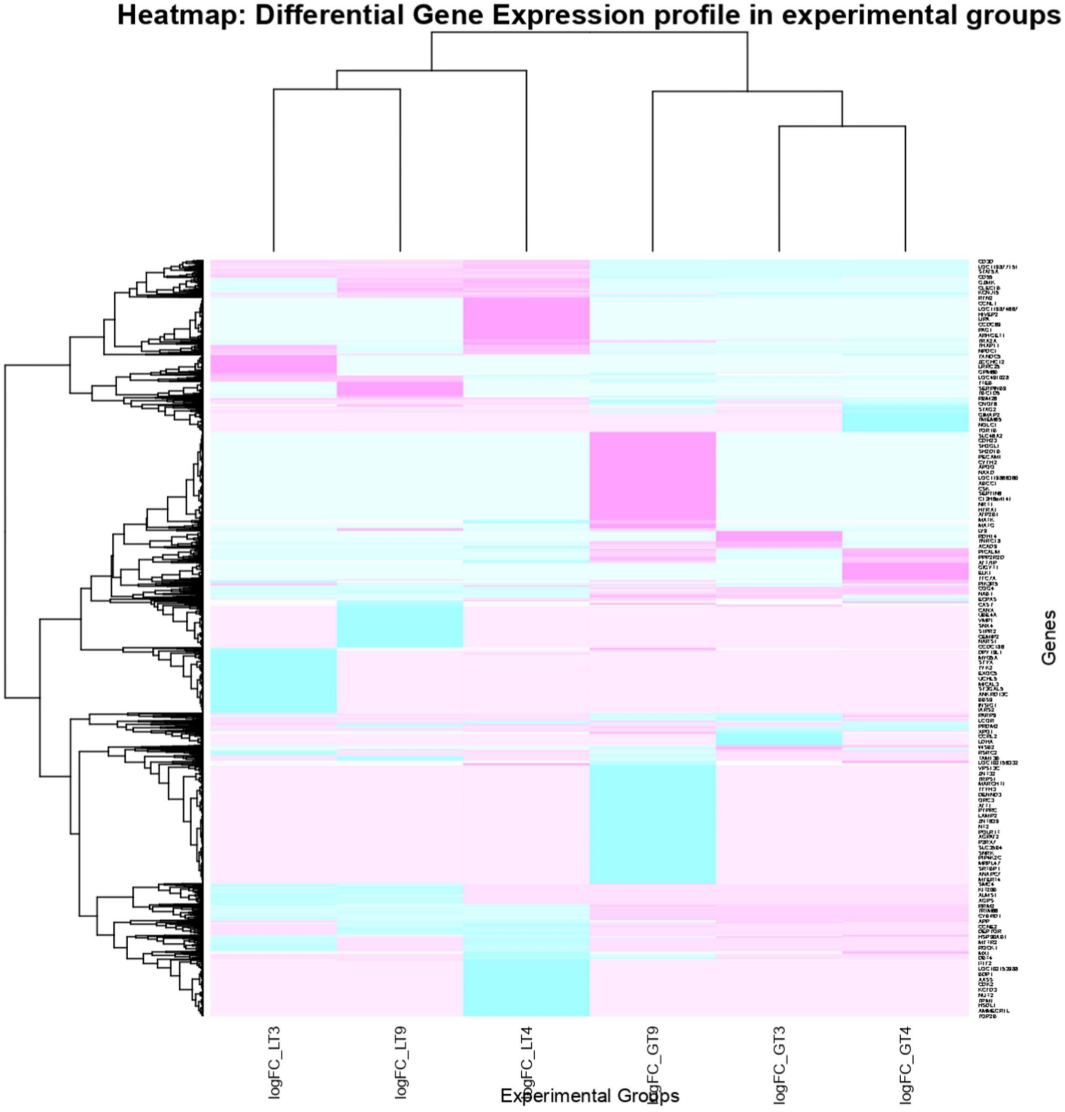
Heatmap of dysregulated DEGs in Labrador and *Gaddi* dog PBMC across all the treatment groups. The heatmap was drawn with the differentially expressed genes (DEGs) with logFC ≥ or ≤−3 and *p*-value < 0.05.

Venn diagram analysis (Figure 2) of the DEGs in the six experimental groups of Labrador and Gaddi dogs under various treatments depicted distinct and overlapping gene expression patterns. In Labrador, the number of unique DEGs in the Poly I: C treated group was 227, while in the LPS treated group, 108, and CpG ODN treated was 284. In Gaddi dogs, the number of unique DEGs in the Poly I: C-treated group was 59, 12 unique DEGs were in the LPS-treated group, and 4 in the CpG ODN-treated group. Volcano plots and Heatmap were constructed using an R environment Package focusing on DEGs with logFC ≥ 3 or ≤−3 and a *p*-value < 0.05 for the dog breed across all the treatment groups shown in Figures 3–6.

#### 3.1.2 GO function and KEGG pathway analysis of DEGs in Labrador PBMCs across all the treatments

To further explore insights through GO, the DEGs across the treatment groups were analyzed for functional enrichment. Several upregulated pathways were identified in the Control vs. Poly I: C group of Labrador (Supplementary Table 6). The pathways included Hematopoietic Cell Lineage (CD2, CD8B), T-cell Leukemia Virus 1 Infection (STAT5A, IL2RB), Th1 and Th2 Cell Differentiation (STAT5A, CD3G), and T Cell Receptor Signaling (ITK, CD3G). Pathways related to antiviral defenses, such as Epstein–Barr Virus Infection (DLA-DOA, B2M) and Measles (STAT5A, CD3G). Downregulated DEGs (Supplementary Table 7) in the same group were enriched in pathways linked to reduced metabolic and cellular activities, such as Metabolic Pathways (ACACA, SCD), Cell Cycle (CDK1, MDM2), and Regulation of Actin Cytoskeleton (ROCK1, MYH10). Signaling and protein degradation were altered in pathways like cGMP-PKG signaling and ubiquitin-mediated proteolysis (GUCY1B1, MDM2).

In the Control vs. LPS group, upregulated DEGs (Supplementary Table 8) were used. The pathway enriched in “Pathways in Cancer” with 15 genes, including ITGB1, STAT5A, and EGF. The “Regulation of actin cytoskeleton” pathway involves 13 genes, including ITGB1, CYFIP2, and RAC1. The “Th1 and Th2 cell differentiation” and “Th17 cell differentiation” pathways indicated a strong association with T-cell differentiation and regulation of immune response—first, the “PI3K-Akt signaling pathway” genes, including ITGB1, GHR, and EGF. Most downregulated DEGs (Supplementary Table 9) were related to pathways like the “Efferocytosis” pathway, including PTGER4, CEBPB, and ALOX15 genes. The “Cell cycle” pathway, including CDC20, DBF4, and CDK2, confirms the effect on cell division and cell proliferation. Moreover, it also includes the “Endocytosis” pathway with genes like RAB11FIP1, TFRC, and RAB5C. Finally, the “NOD-like receptor signaling pathway,” including HSP90AB1, CXCL8, and MAPK14. Lastly, the “Cellular senescence” pathway, with genes like CXCL8, CDK2, and MAPK14.

The Upregulated DEGs (Supplementary Table 10) in the Control vs. CpG group were significantly enriched in biological pathways, including “Ribosome” with genes such as RPS5, RPLP0, and RPL37A. “Pathways in cancer” pathway, represented by genes ITGB1, STAT5A, and EGF. The “Hematopoietic cell lineage” pathway includes CD2, CD8B, and CD3G. Furthermore, “Th1 and Th2 cell differentiation” and “Th17 cell differentiation” pathways involve genes like STAT5A, IL2RB, and RELA. The downregulated DEGs (Supplementary Table 11) and the Control vs. CpG DEGs were associated with the “Cell cycle”: CDC20, CCNE2, CDK1. Pathway “Lipid and Atherosclerosis” and related genes, for example, LYN, SELP, OLR1. For example, genes such as RBL1, CCNE2, and MDM2. “Transcriptional misregulation dysregulation in cancer” shows genes such as NCOR1 and MDM2. The “PPAR signaling pathway” encompasses genes including FADS2 and ACSL5.

#### 3.1.3 GO function and KEGG pathway analysis of DEGs in Gaddi dog PBMCs across all the treatments

The differential gene expression analysis identified 204 dysregulated genes in Gaddi dogs following the Contol vs. Poly I: C treatment, with 112 downregulated and 92 upregulated. In the Poly I: C-treated Gaddi dog group, several upregulated (Supplementary Table 12) DEGs are linked to biological pathways and immune responses. The identified DEGs involve cancer, RNA metabolism, viral infections, and immune cell differentiation pathways. For instance, genes like NOTCH2, IFNGR2, and MDM2 are upregulated in the Pathways in Cancer—specifically, NOTCH2 and IFNGR2. Additionally, the Metabolism of the RNA pathway shows the upregulation of genes like HNRNPA3 and XPO1. The Th1 and Th2 cell differentiation pathways include upregulated genes like JAK2. The presence of upregulated genes in pathways such as Epstein–Barr Virus Infection and Influenza A, including XPO1 and IFNGR2, points to the activation of broad antiviral mechanisms in the Gaddi dog’s immune system—furthermore, pathways like mRNA Splicing. While the downregulated (Supplementary Table 13) DEGs in the Control vs. Poly I: C treated group of Gaddi dogs showed pathways highlighting, such as in cancer with NOTCH2, MDM2, and E2F3, the pathway related to RNA metabolism was also enriched for genes such as HNRNPA3 and XPO1. On the other hand, endocrine resistance and prostate cancer pathways are highlighted by genes such as MDM2 and LEF1, Oncogene-induced senescence, and MDM2 and ETS1. Negative regulation of gene expression and transcription by RNA polymerase II, Membrane Trafficking, Vesicle-mediated Transport, T Cell Receptor Signaling, and Efferocytosis pathways involving genes like SNAP23, LCP2, ICOS, and NFATC2. Downregulated genes such as NCK1, TNFAIP3, and JAK2 were linked to T-cell activation, Toll-like receptor signaling, and inflammation pathways.

The differential expression analysis in the Control vs. LPS group of Gaddi dogs revealed 354 DEGs, with 185 significantly upregulated and 149 downregulated. In the Control vs. LPS-group Gaddi dog group, several upregulated DEGs (Supplementary Table 14) highlight the activation of immune pathways and cellular processes. The Immune System pathway has many upregulated genes, including CD274, STAT3, NLRP3, and LCP2, which are crucial for modulating immune responses, inflammation, and cellular signaling. CD274. Further, the Signaling by Rho GTPases pathways shows upregulation of genes such as SNAP23 and PTK2B. The Neutrophil Degranulation pathway features upregulated genes like CXCR2, MMP8, and LTF the Adaptive Immune System pathway, genes such as HECTD3 and LCP2. Additionally, Interleukin-4 and Interleukin-13 Signaling include upregulated STAT3 and SOCS5, suggesting a shift toward Th2-type immune responses. In the Control vs. LPS Gaddi dog group, the downregulated DEGs (Supplementary Table 15) indicate a reduction in various immune responses and transcriptional activities. The Immune System pathway shows significant downregulation of key genes like PYCARD, RELA, NFKB1, and SOCS5. These genes are central to inflammatory signaling and immune regulation, with RELA and NFKB1 being crucial components of the NF-κB pathway. Downregulating genes, like CNOT6, ELF1, TAF11, and POLR2G, indicate a broader suppression of transcriptional activity in the RNA Polymerase II Transcription and Gene Expression pathways. These genes are involved in initiating and regulating transcription, and their decreased expression may be linked to the need to control excessive cellular activity during inflammatory responses—downregulation of genes such as ADGRE5 and SLAMF7. Furthermore, downregulation in pathways like CLEC7A (Dectin-1) Signaling and C-C-type lectin Receptors (CLRs) includes genes like PYCARD, RELA, and NFKB1.

The differential gene expression analysis between the control vs. CpG treatment group in Gaddi dogs identified 1,072 DEGs, with 506 upregulated and 566 downregulated. Upregulated DEGs (Supplementary Table 16) are enriched in several pathways: PI3K-Akt signaling (e.g., CSF3, PIK3R1, BCL2L1), NOD-like receptor signaling (e.g., GSDMD, RIPK2), and MAPK signaling (e.g., MAPK14, MAPK8), Other pathways include Chemokine signaling (e.g., CCL22, CXCR2), Ras signaling (e.g., RAB5C, RAP1B), The Cytokine-cytokine receptor interaction pathway highlights CSF3 and L1R2 is essential for immune cell communication. In contrast, FoxO signaling and Neurotrophin signaling were also seen. The Downregulated DEGs (Supplementary Table 17), the enriched pathways in Control vs. CpG treated Gaddi dog samples. The “Pathways in Cancer” involve ITGB1, NOTCH2, and HSP90AB1 genes. The “Salmonella Infection” pathway includes HSP90AB1, IRAK4, and PIK3C2A, focusing on host-pathogen interactions and inflammation. In the “Influenza A” pathway, genes like MX2, STAT2, and CXCL10 contribute to antiviral responses. The “Lipid and Atherosclerosis” pathway highlights HSP90AB1, IRAK4, and JAK2, which are associated with lipid metabolism and immune responses. The “MAPK Signaling Pathway” includes genes like MAP3K2, PLA2G4A, and NFKB1, Lastly, the “Tuberculosis” pathway involves IFNGR2, ITGB2, and IL12B.

In the study, six DEGs (Table 5) shared between Labrador and Gaddi dogs are ITK, IL2RB, CD3E, CD3G, FCGRT, and SLPI. ITK—Inducible T Cell Kinase—is a CD3E and CD3G component of the T cell receptor complexes. The gene FCGRT encodes the Fc gamma receptor and transporter. Finally, SLPI -Secretory Leukocyte Peptidase Inhibitor, a protease inhibitor with anti-inflammatory modulating activity. Moreover, the Protein–protein Interactions were drawn using the STRING tool for Labrador and Gaddi dog PBMC across all the groups as shown in Supplementary Figures 1, 2, respectively.

**Table 5:** All the immune system genes common across the experimental group in Labrador.

S. No.	Common genes
1	ITK (IL2 Inducible T Cell Kinase)
2	IL2RB (Interleukin 2 Receptor Subunit Beta)
3	CD3E (CD3 Epsilon Subunit Of T-Cell Receptor Complex)
4	CD3G (CD3 Gamma Subunit Of T-Cell Receptor Complex)
5	FCGRT (Fc Gamma Receptor and Transporter)
6	SLPI (Secretory Leukocyte Peptidase Inhibitor)

Green highlight represents upregulated genes. Red highlight represents a down-regulated gene.

### 3.2 miRNA Seq and data summary

The miRNA sequencing of eight Labrador and Gaddi dog samples using the Illumina NovaSeq 6,000 platform produced detailed metrics. Quality assessment was performed using FastQC and MultiQC, followed by preprocessing with Trimgalore and Cutadapt to remove adapters and low-quality bases. The preprocessed reads were aligned to the *Canis lupus familiaris* reference genome (ROS_Cfam_1.0; GCF_014441545.1) using the miRDeep2 mapper.pl. script, ensuring accurate miRNA mapping. The alignment results revealed distinct read-mapping patterns are shown in Tables 3. Among Labrador dogs, the Labrador-Control sample had 6.8 million mapped reads, demonstrating high mapping efficiency, while Poly I:C and LPS samples showed slightly lower reads at 6.6 million and 6.4 million, respectively. The CpG sample had the lowest mapped reads (3.6 million), indicating a potential effect of CpG treatment on alignment. In Gaddi dogs, the Control sample had the highest mapped reads at 8.3 million, indicating strong alignment, while Poly I:C and LPS samples showed moderate decreases with 3.8 million and 6.3 million reads, respectively. The Known and Novel miRNAs identified for Labrador and *Gaddi* dogs PBMCs across the treatment groups using miRDeep2 as statistics represented in Table 6.

**Table 6:** Known and novel miRNAs identified for labrador and *Gaddi* dogs PBMCs across the treatment groups using miRDeep2.

S. No.	Sample ID	Expressed miRNAs	Known miRNAs	Novel miRNAs
1	L-Control	2,025	185	1,840
2	L-Poly IC	2,248	154	2,094
3	L-LPS	2,068	191	1,877
4	L-CpG	1,946	190	1,756
5	G-Control	1,259	194	1,065
6	G-Poly IC	1,011	190	821
7	G-LPS	176	33	143
8	G-CpG	829	180	649

This colour gradient helps to highlight patterns or contrasts in the dataset and emphasizes biologically or statistically significant trends in data set.

#### 3.2.1 Identification of differentially expressed miRNAs and target gene prediction

**Figure 7:**
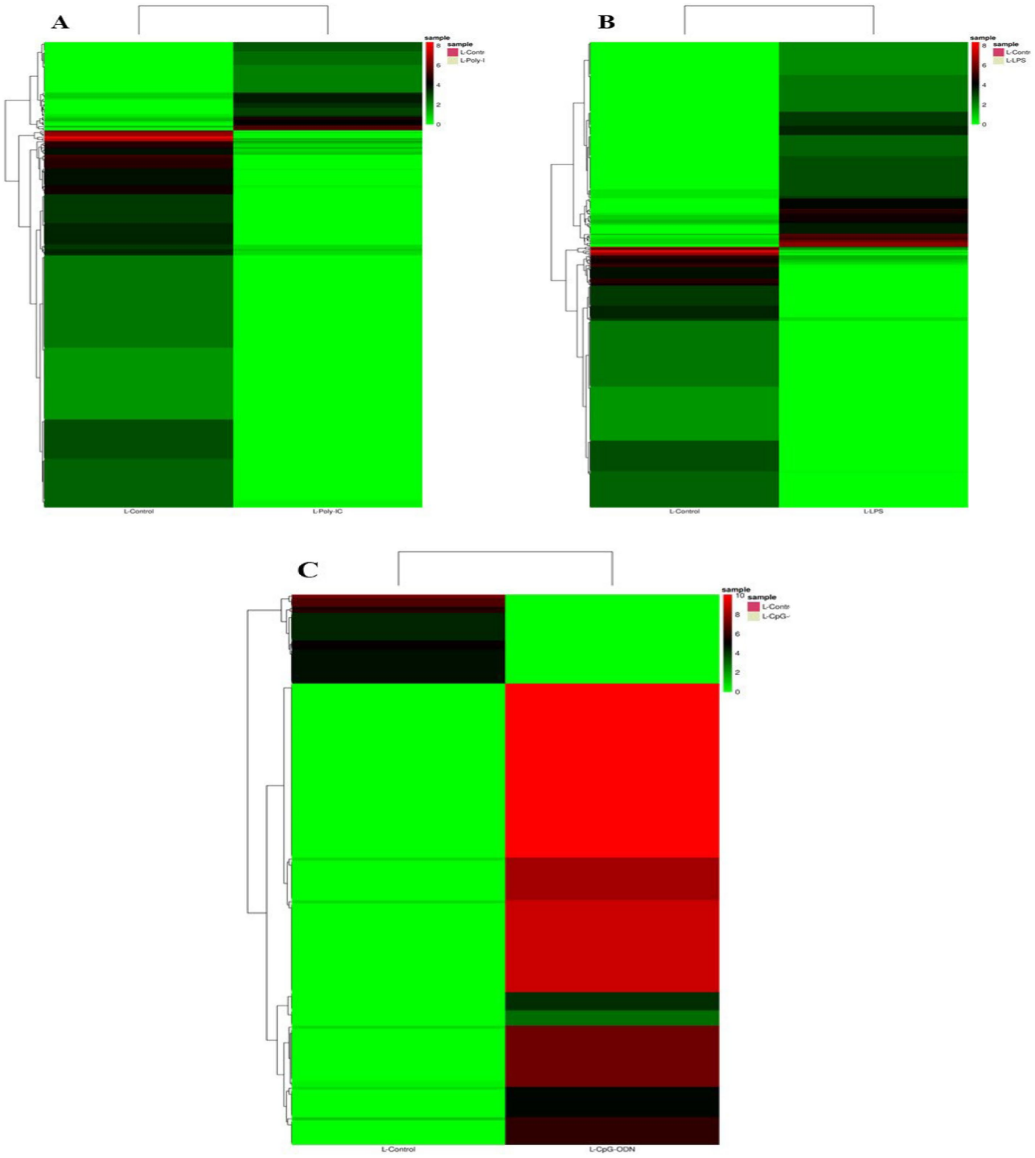
Expression profile of the significant differentially expressed miRNAs across all the treatment groups in Labrador dog. **(A)** Ctrl vs. Poly IC miRNA, **(B)** Ctrl vs. LPS miRNA, **(C)** Ctrl vs. CpG miRNA. The heatmap is created by calculating a z-score for each row from the normalized read counts of the samples.

**Figure 8:**
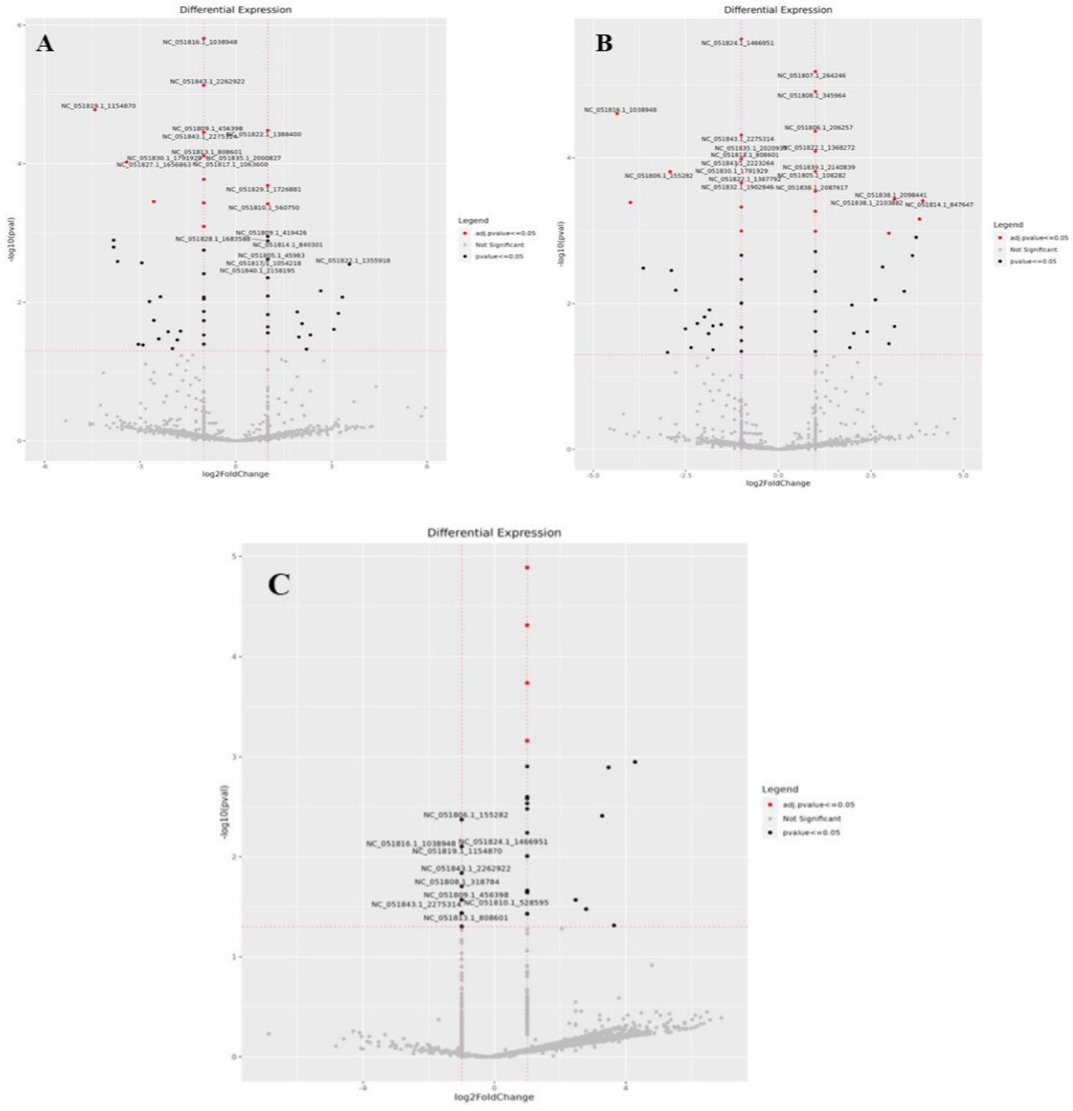
Volcano plot showing differential expression profile of miRNAs across all the treatment groups of Labrador dog. **(A)** Ctrl vs. Poly IC miRNA, **(B)** Ctrl vs. LPS miRNA, **(C)** Ctrl vs. CpG miRNA. Black indicates log2 fold change≥1 and *p* value≤0.05. “Red” dots indicate absolute log2 fold change≥1 and FDR/adjusted *p* value≤0.05.

**Figure 9:**
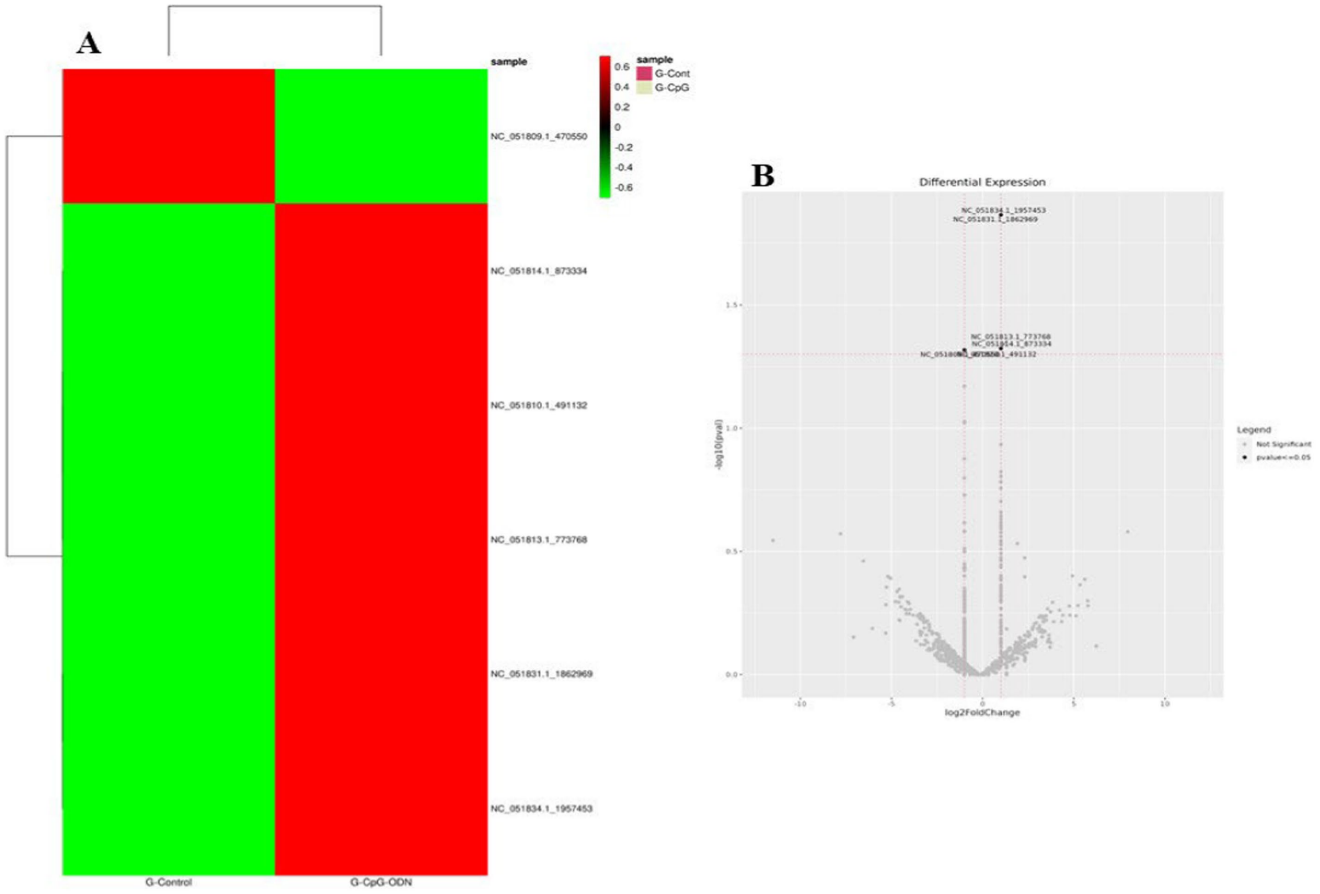
**(A)** Expression profile of the significant differentially expressed miRNAs in Ctrl vs. CpG treatment group in *Gaddi* dog. **(B)** Volcano plot showing differential expression profile of miRNAs in Ctrl vs. CpG treatment group in *Gaddi* dog. Black indicates log2 fold change≥1 and *p* value≤0.05. “Red” dots indicate absolute log2 fold change≥1 and FDR/adjusted *p* value≤0.05.

Differential expression analysis was performed using DESeq2 v1.34.0, applying variance stabilizing transformation for normalization. miRNAs with an absolute log2 fold change ≥ 1 and *p*-value ≤ 0.05 were considered significant, as shown in Table 7. The heatmap and Volcano plots of differentially expressed miRNA in Labrador and Gaddi dog PBMCs were constructed using R-programming environments as shown in Figures 7–9 and followed by the target gene prediction of miRNAs in Labrador and Gaddi dogs across all the treatment groups using three online tools, including TargetScan,^8^ miRDB,^9^ and MirTarBase.^10^

**Table 7:** Differentially expressed miRNAs in simulated PBMCs of Labrador and *Gaddi* dogs PBMCs across the treatment groups.

Comparison	Total miRNAs	Differentially expressed miRNAs	Significantly expressed miRNAs	Up regulated miRNAs	Down regulated miRNAs
Gr-1 vs. Gr-2	3,957	2,416	306	58	248
*L-Ctrl-Poly IC*					
Gr-1 vs. Gr-3	3,957	2,504	396	174	222
*L-Ctrl-LPS*					
Gr-1 vs. Gr-4	3,957	3,098	180	151	29
*L-Ctrl-CpG*					
Gr-5 vs. Gr-6	3,957	1,457	0	0	0
*G-Ctrl-Poly IC*					
Gr-5 vs. Gr-7	3,957	1,326	0	0	0
*G-Ctrl-LPS*					
Gr-5 vs. Gr-8	3,957	1,441	6	5	1
*G-Ctrl-CpG*					

This colour gradient helps to highlight patterns or contrasts in the dataset and emphasizes biologically or statistically significant trends in data set.

#### 3.2.2 GO function and KEGG pathway analysis of DE target genes in Labrador PBMCs across all the treatments

**Figure 10:**
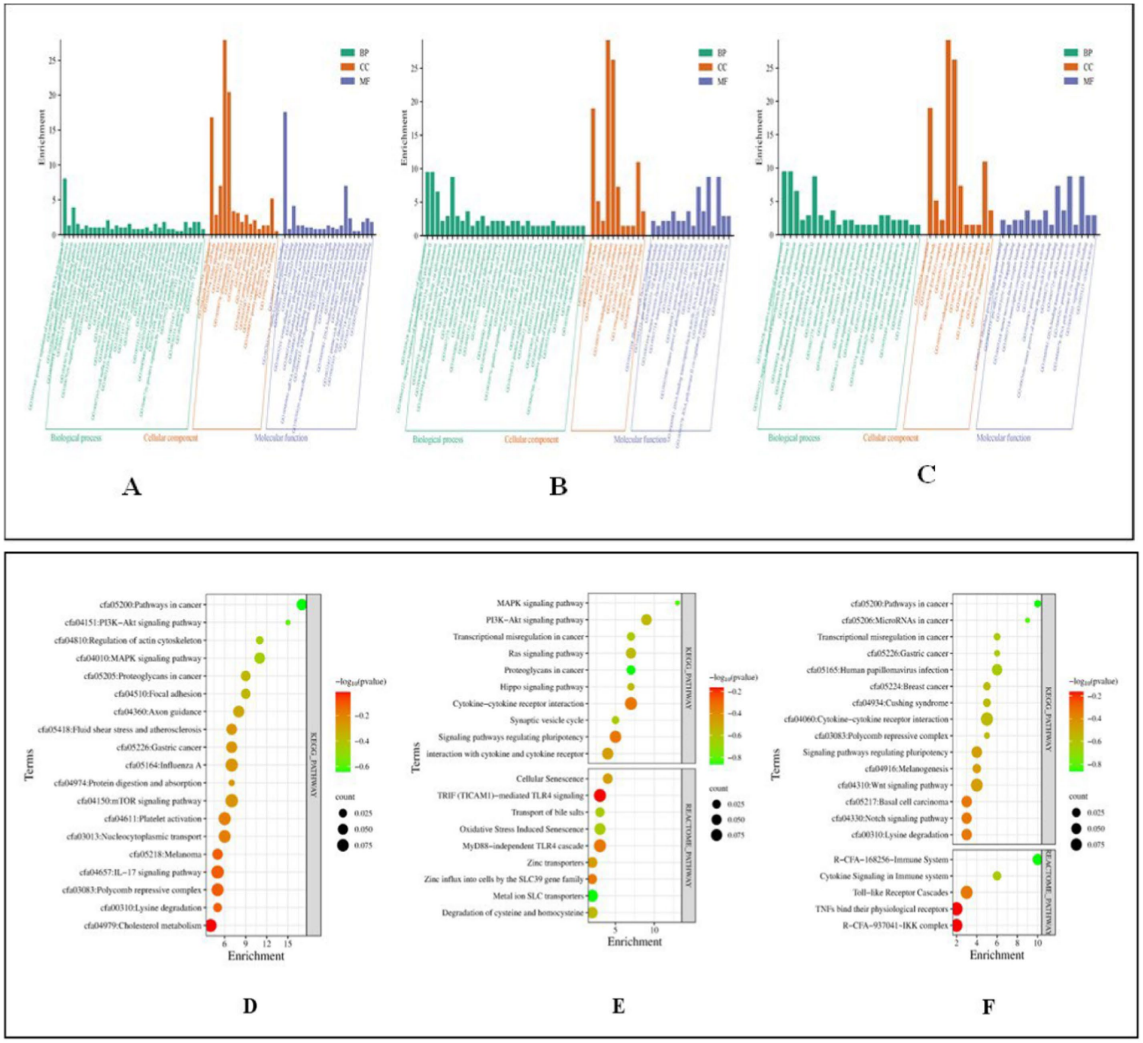
Functional classification of miRNA targets of Labrador dog based on gene ontology (GO) terms (david.ncifcrf.gov/) and Dot plot showing enriched KEGG pathway (www.genome.jp/kegg/pathway.html). **(A)** GO terms of control vs. PolyIC targets. **(B)** GO terms for control vs. LPS targets. **(C)** GO terms for Control vs. CpG targets. **(D)** Pathway enriched in control vs. PolyIC targets. **(E)** Pathway enriched in control vs. LPS targets. **(F)** Pathway enriched in control vs. CpG targets. The pathways are represented on the y-axis, while the x-axis indicates the enrichment score. The color gradient represents the -log10(*p*-value), where red indicates higher significance (lower *p*-value) and green indicates lower significance (higher *p*-value). The size of the dots represents the count of genes involved in each pathway. Representation of functional enrichment was created from an online tool (www.bioinformatics.com.cn/en).

Functional analysis of miRNA targets from Control vs. Poly I:C Labrador PBMCs identified 478 targets involved in diverse biological processes, cellular components, and molecular functions. Biological processes included positive-regulation of transcription by RNA polymerase II, gene expression, and cellular response to hydrogen peroxide. Important cellular components were nucleoplasm, nucleus, and cytosol, reflecting their involvement in fundamental cellular structures. Analysis of 385 miRNA targets from Control vs. LPS in Labrador PBMCs revealed GO terms. Key biological processes included positive regulation of gene expression, transcription by RNA polymerase II, cell differentiation, and mRNA splicing via the spliceosome. Fibroblast growth factor receptor signaling, cytokine signaling, B-cell differentiation, and the canonical Wnt signaling pathway were notable pathways involved. Major cellular components include the nucleus, nucleoplasm, cytoplasm, and extracellular space. Lastly, in the control vs. CpG group of Labrador-PBMCs, miRNA 423 target genes were significantly enriched in various biological processes and molecular functions. These miRNAs played crucial roles in the positive and negative regulation of gene expression and transcription by RNA polymerase II. They were also involved in cell differentiation processes, such as fat cell and B cell differentiation shown in Figure 10.

#### 3.2.3 GO function and KEGG pathway analysis of DE target genes in Gaddi dogs PBMCs across all the treatments

**Figure 11:**
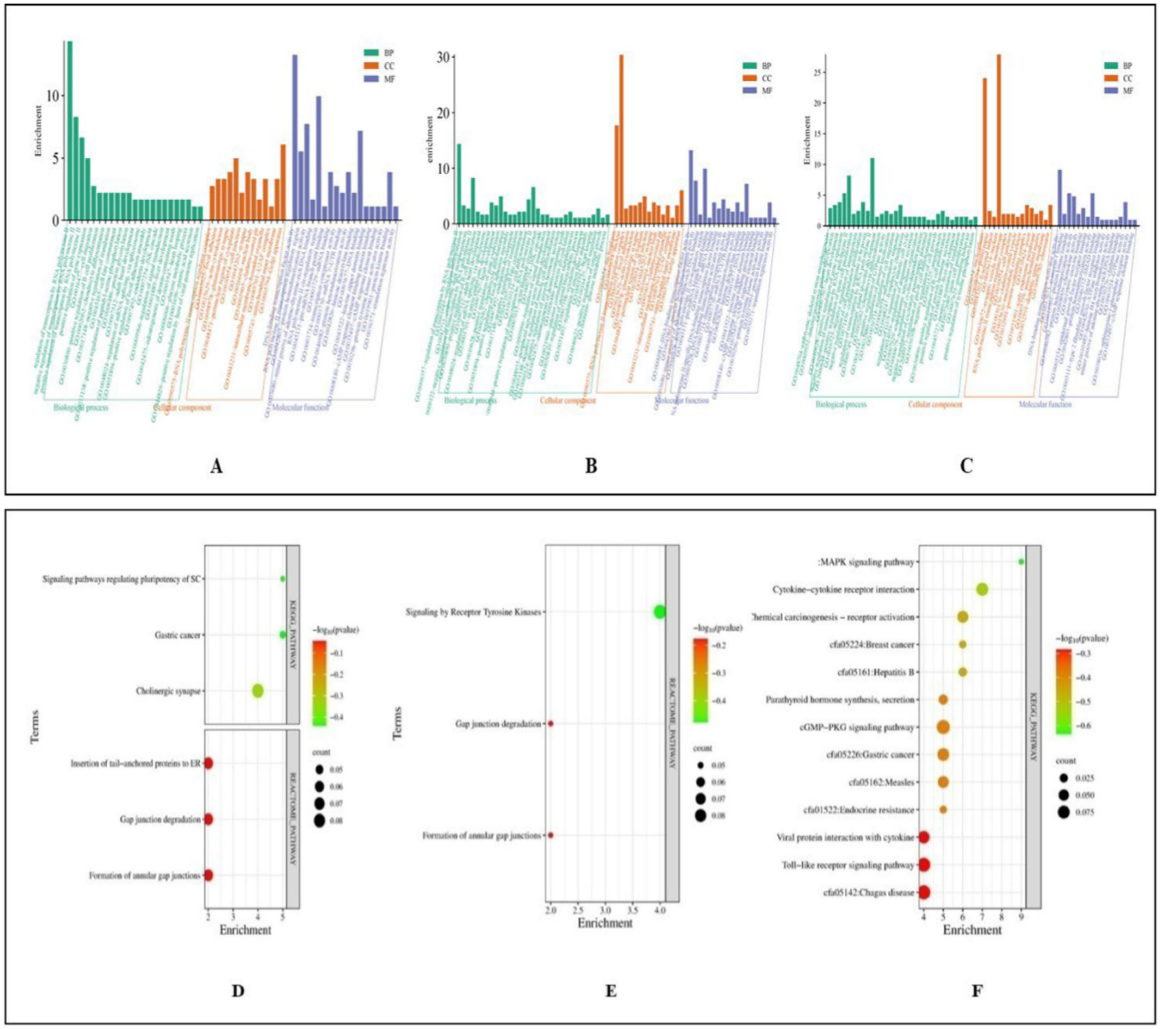
Functional classification of miRNA Targets of *Gaddi* dogs based on gene ontology (GO) Terms (david.ncifcrf.gov/) and dot plot showing enriched KEGG pathway (www.genome.jp/kegg/pathway.html). **(A)** GO terms of control vs. PolyIC targets. **(B)** GO terms for control vs. LPS targets. **(C)** GO terms for control vs. CpG targets. **(D)** Pathway enriched in control vs. PolyIC targets. **(E)** Pathway enriched in control vs. LPS targets. **(F)** Pathway enriched in control vs. CpG targets. The pathways are represented on the y-axis, while the x-axis indicates the enrichment score. The color gradient represents the -log10(*p*-value), where red indicates higher significance (lower *p*-value) and green indicates lower significance (higher *p*-value). The size of the dots represents the count of genes involved in each pathway. Representation of functional enrichment was created from an online tool (www.bioinformatics.com.cn/en).

Functional analysis of 385 miRNA targets in Control vs. Poly I:C Gaddi dog PBMCs revealed significant GO terms biological processes, including regulation of transcription by RNA polymerase II, cell migration, positive regulation of B cell proliferation, and protein glycosylation. Cellular components highlighted were the RNA polymerase II transcription regulator complex, focal adhesion sites, actin cytoskeleton, glutamatergic synapse, and perinuclear region of the cytoplasm. Molecular functions showed enrichment in DNA-binding transcription factor activity, RNA polymerase II transcription regulatory region binding, and protein homodimerization. Immune-related terms included positive regulation of B cell proliferation and the JNK cascade. In Control vs. LPS Gaddi dog PBMCs, GO analysis of 287 miRNA targets revealed enrichment in the regulation of transcription by RNA polymerase II, B cell proliferation, protein glycosylation, and cell migration. Cellular components prominently included the nucleus, nucleoplasm, RNA polymerase II transcription regulator complex, and focal adhesion. Molecular functions highlighted DNA-binding transcription factor activity, RNA polymerase II-specific binding, protein homodimerization, and histone deacetylase binding. For Control vs. CpG in Gaddi dogs, analysis of 365 miRNA targets identified key processes like the inflammatory response, B cell proliferation, lymphocyte chemotaxis, and cell migration. Significant cellular components included the nucleoplasm and nucleus. Molecular functions revealed chemokine activity, protein kinase binding, and DNA-binding transcription activator activity, crucial for immune cell signaling and regulation (Figure 11).

#### 3.2.4 Common differentially expressed miRNA between and within Labrador and Gaddi dogs

**Figure 12:**
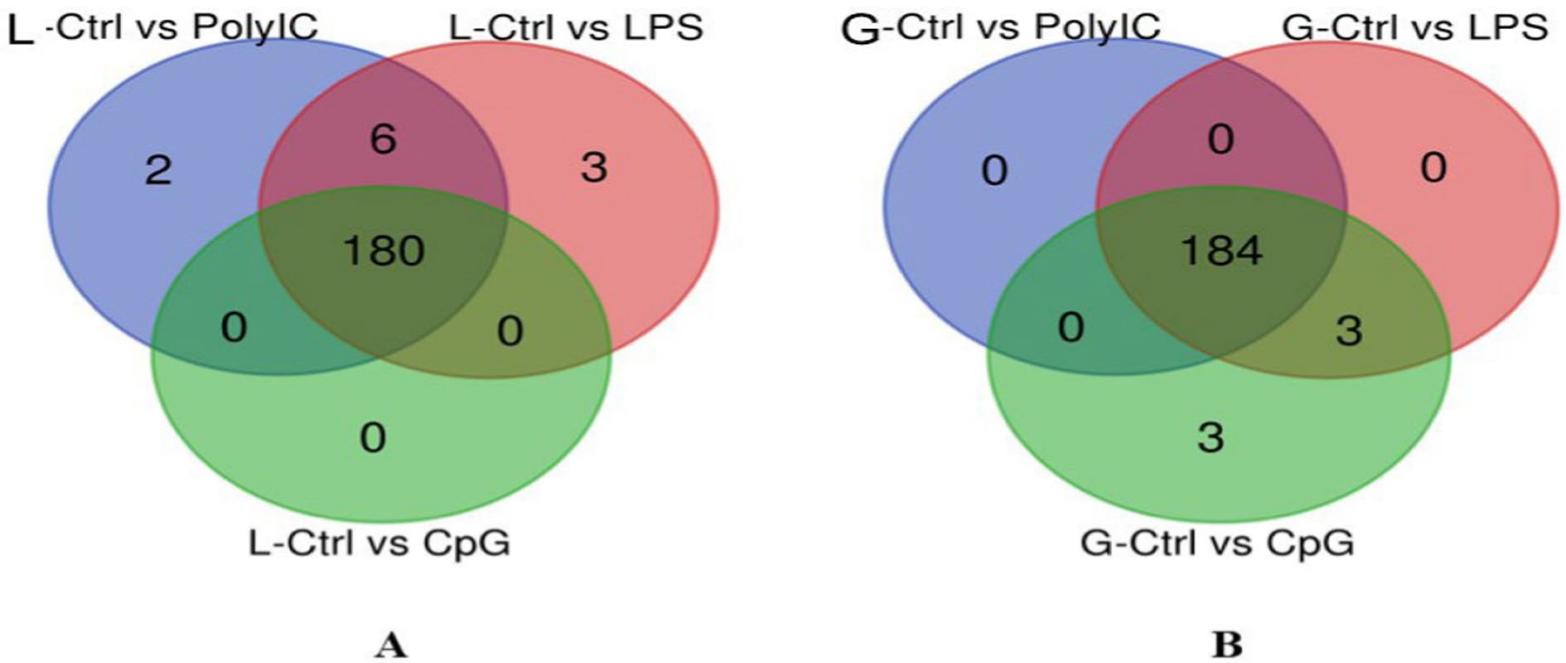
Venn diagram representing the overlapping miRNA within a breed of dog. **(A)** Differentially expressed (DE) miRNAs in Labrador dogs across the three-treatment group. **(B)** Differentially expressed (DE) miRNAs in *Gaddi* dogs across the three-treatment group. The representation of the Venn diagram was created using an online tool (bioinformatics.psb.ugent.be/webtools/Venn/).

The Venn diagram in Figure 12 was constructed to identify the common and unique miRNAs in both Labrador and Gaddi dogs PBMCs across all the treatment groups. In Labrador dog PBMC, overall, 180 miRNAs were commonly shared across three treatment groups. However, six miRNAs (cfa-miR-204, 206, 106a, 132, 335, and 676) were shared between Control vs. LPS and Control vs. Poly I:C. Interestingly, two unique miRNAs (Cfa-miR-196a and 144) were identified in Control vs. Poly I:C. Similarly, 2 were uniquely miRNAs (Cfa-miR-152 and 1) identified in the Control vs. LPS group of Labrador dog PBMCs. In Gaddi dog PBMC, over all 184 miRNAs were shared between all three treatment groups. Moreover, the Control vs. CpG and Control vs. LPS groups shared only three common miRNAs (Cfa-miR-98, 182, and 203). Similarly, the Control vs. CpG group was identified with 3 unique miRNAs (Cfa-miR-449a, 193b, and 206).

**Figure 13:**
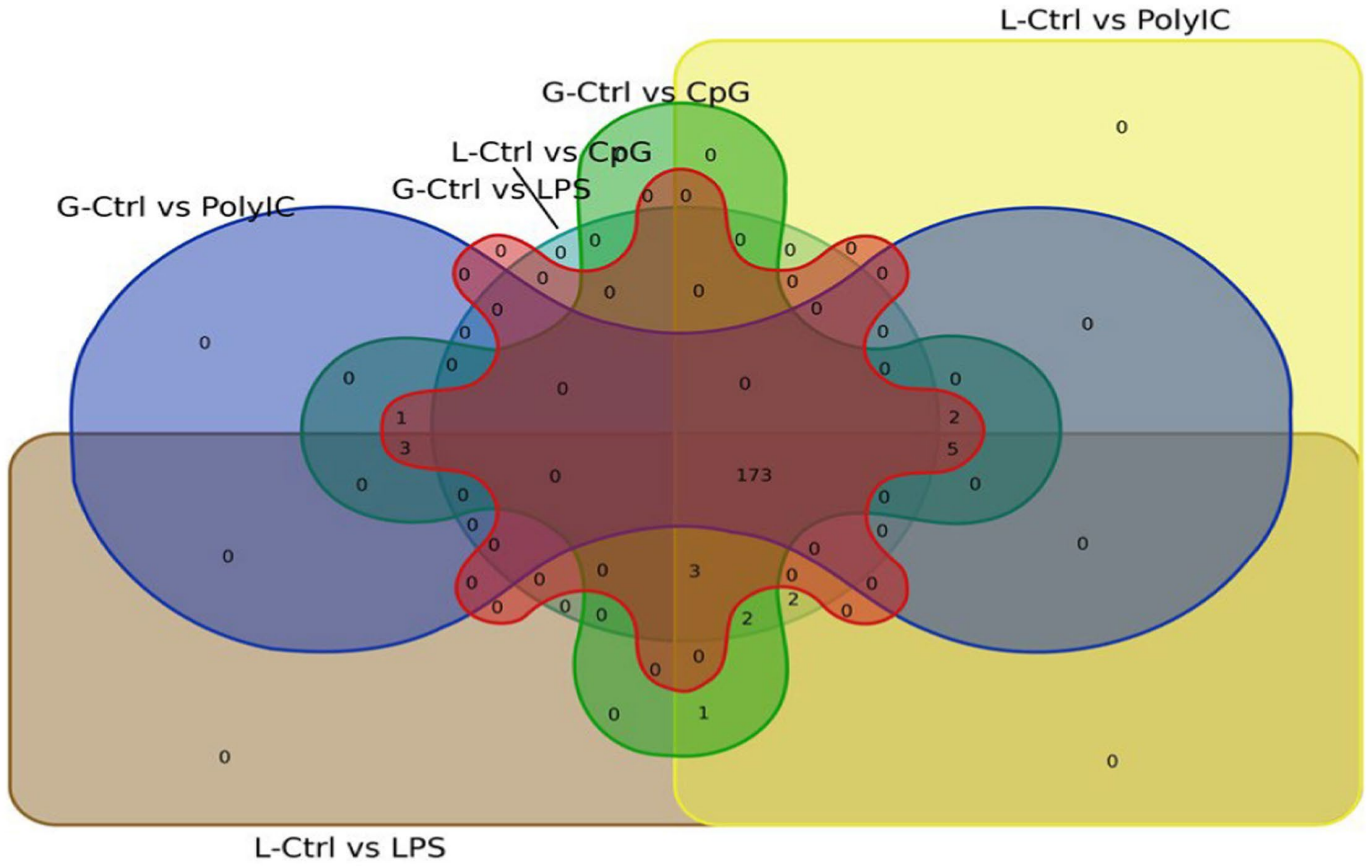
Venn diagram representing the overlapping differentially expressed miRNA between the Labrador dog and *Gaddi* dog across the treatment groups.

In the analysis of differentially expressed miRNAs, a cross-breed comparison was constructed in a Venn diagram (Figure 13). One hundred seventy-five (175) miRNAs across various treatments in Labrador and Gaddi dogs were commonly shared. Surprisingly, Labrador dog PBMC showed two unique miRNAs (Cfa-miR—551a and 1,249) expressed across all the treatment groups.

### 3.3 qPCR validation of mRNA and miRNA Seq

To verify the accuracy of our RNA-seq and miRNA-seq analyses, we selected two upregulated DEGs and two downregulated DEGs that were commonly expressed across all three treatment groups in both Labrador and Gaddi dogs for qPCR validation. Additionally, we selected one upregulated DEG common across all treatment groups in Labrador and three downregulated DEGs specific to each treatment group in Labrador. For Gaddi dogs, we included two upregulated DEGs common to two and one treatment, respectively, and groups, respectively, and one downregulated DEG common across all treatment groups. The qPCR results showed expression levels consistent with those observed in our RNA-seq and miRNA-seq analyses. In Labrador PBMCs, PPP1R12A was significantly downregulated (*p* < 0.05), linked to immune cell transmigration and apoptosis (34). SLMAP was upregulated considerably (*p* < 0.05), emphasizing its role in cell migration and immune regulation (35). In Gaddi PBMCs, WSB2 was significantly upregulated (*p* < 0.05), impacting I21 receptor regulation and immune responses (36), while ATP6AP2 was downregulated but not significantly (*p* > 0.05) (Figure 13). In Labrador dogs, qPCR results showed ARMC8 significantly upregulated (*p* < 0.05) across all treatment groups, highlighting its role in innate immunity and neutrophil degranulation. FIBCD1 was significantly downregulated (*p* < 0.05) in the control versus Poly I:C group (37). VPS13D and STARD13, involved in apoptosis and lymphocyte proliferation, were significantly downregulated (*p* < 0.05) in the control versus LPS and control versus CpG groups, respectively (38), as depicted in Supplementary Figures 2–4, confirming the accuracy and reliability of our transcriptomic data.

## 4 Discussion

India possesses a rich diversity of Indigenous dog breeds, which have evolved in tandem with local climatic and environmental conditions over time (2). Numerous studies highlight the disease resistance and robust immune responses exhibited by Indigenous animals. According to (39), native breeds generally exhibit higher disease resistance than non-native or exotic breeds due to their adaptation to local environmental stressors (40). This resilience is primarily attributed to genetic traits shaped by natural selection, enabling greater tolerance to pathogens and reduced dependence on intensive management (41). Gaddi dogs, a free-ranging breed traditionally used by the Gaddi tribe for livestock guarding in mountainous regions, are pre-adapted to harsh climatic conditions and high altitudes. Such adaptive traits may confer advantages in human-altered environments through hybridization, potentially enhancing immunity against environmental pathogens (4). The official recognition of the Gaddi dog breed by the National Bureau of Animal Genetic Resources (NBAGR; See footnote 1; Accession number INDIA_DOG_0600_GADDI_19004) marks a significant step toward preserving and promoting this indigenous breed. Recognition validates its distinct genetic identity, ensuring focused conservation efforts, and breeding programs. Investigating Gaddi dogs’ transcriptomic and miRNAome profiles and comparing them with the widely owned Labrador Retriever has yielded valuable insights contributing to the existing knowledge base.

Cultured PBMCs were divided into three treatment groups to simulate bacterial and viral infections, along with a control group. During the treatment period, the PBMCs in the stimulated groups showed rapid division and dense growth, while the control group exhibited a lower cell count. The cells were further processed for mRNA and miRNA extraction. The mRNA and miRNA sequencing of Labrador and Gaddi dog PBMCs simulated with various TLR ligands {Control vs. Poly I:C (TLR-3), Control vs. LPS (TLR-4), and Control vs. CpG ODN(TLR-9)} was done using Illumina NovaSeq-6000 platform. Raw reads were generated, and considering the dog genome size of approximately 2.5 GB (82) with a sequencing depth of 15x, it was estimated that 5–10% of the cellular RNA is coding RNA (mRNA). Thus, the expected raw data per sample was around 4 GB. However, the Gaddi dog PBMCs in the Control, Poly I:C, and CpG groups produced 12.13 GB, 11.98 GB, and 9.17 GB of data, respectively (Table 2). In the differential expression analysis across Labrador and Gaddi dog groups, distinct patterns emerged. For Labradors, significant DEGs were observed in all comparisons, with the Ctrl vs. Poly I:C group revealing 5,476 significant DEGs out of 18,954 total, the Ctrl vs. LPS group showing 5,621 significant DEGs out of 19,381, and the Ctrl vs. CpG ODN group identifying 4,978 significant DEGs out of 19,822. In Gaddi dogs, the Ctrl vs. Poly I:C comparison identified 1,482 significant DEGs out of 22,251 total, the Ctrl vs. LPS group had 3,645 significant DEGs out of 22,800, and the Ctrl vs. CpG ODN group showed 2,416 significant DEGs out of 22,463. The variation in DEG counts between the breeds highlights differences in their immune responses to the same stimuli, indicating breed-specific molecular mechanisms.

The mRNA Seq analysis in Labrador dog PBMCs across all the treatments has shown common enrichment of Th1 and Th17 terms, and the interplay between them is associated with signaling pathways that are prominent in immune-related response (42). In the Control vs. Poly I:C group of Labrador-, The Hematopoietic Cell Lineage pathway showed an increase in genes like CD2 and CD8B, suggesting boosted development and function of immune cells. A study by (43) is a cell adhesion molecule primarily expressed on T cells and natural killer (NK) cells, where it interacts with CD58 (LFA-3) on antigen-presenting cells (APCs). The Human T-cell Leukemia Virus 1 Infection pathway involved genes such as STAT5A and IL2RB, highlighting a heightened immune response against viral infections. Th1 and Th2 Cell Differentiation and Th17 Cell Differentiation pathways, featuring genes like STAT5A and CD3G, pointed to enhanced specific immune responses (44–46). Zhang et al. (47) highlighted the critical regulation of Th1 and Th2 cells, emphasizing that any imbalance in their function could contribute to various immunopathological conditions, including allergies, immune deficiencies, and lymphomas. The CD3E gene is vital for TCR-CD3 complex assembly, stability, and surface expression, impacting T-cell proliferation and cytokine production. These genes are involved in crucial pathways such as TCR signaling, PD-L1/PD-1 checkpoint pathways, and hematopoietic cell lineage, emphasizing their role in immune homeostasis and immune cell development. CD3E deficiencies can cause severe immunodeficiencies, demonstrating its importance in maintaining immune function. Their involvement in checkpoint pathways highlights their relevance in cancer immune evasion (78). Understanding CD3E and CD3G functions offers insights into therapeutic targeting in immune-related diseases and cancer. Further research could reveal strategies for modulating immune responses in conditions with compromised T-cell function (48). Moreover, the ITK gene plays a pivotal role in several key immune pathways, particularly the T-cell receptor (TCR) signaling pathway, chemokine signaling pathway, and leukocyte trans endothelial migration. In the TCR signaling pathway, ITK is activated upon TCR engagement and recruited to the plasma membrane, where it phosphorylates phospholipase C gamma 1 (PLCγ1). This phosphorylation generates secondary messengers such as DAG and IP3, initiating downstream signaling cascades involving MAPK, NF-κB, and calcium signaling, ultimately driving cytokine production, T-cell survival, proliferation, and differentiation (49, 50). ITK also influences the chemokine signaling pathway, critical for T-cell migration and homing. Its activity supports cytoskeletal rearrangement and cell adhesion, promoting efficient chemotaxis to sites of infection and inflammation. Additionally, ITK is integral to leukocyte transendothelial migration, regulating the signaling required for leukocytes to traverse the endothelial barrier into tissues. This process is vital for immune surveillance and response, as ITK coordinates with other molecules to facilitate proper leukocyte adhesion and transmigration (51). The IL2RB gene plays a central role in immune regulation, participating in pathways such as Cytokine-Cytokine Receptor Interaction, Viral Protein Interaction with Cytokine and Cytokine Receptor, Endocytosis, PI3K-Akt Signaling, JAK-STAT Signaling, and Th1/Th2 Cell Differentiation. These pathways highlight its importance in both routine immune function and pathogen response. IL2RB is particularly crucial for Th1 and Th2 cell differentiation, influencing whether the immune response is cell-mediated (Th1) or humoral (Th2). Additionally, its involvement in the JAK–STAT pathway underscores its significance in cytokine responses, vital for immune function and inflammation control (52). The FCGRT gene encodes the neonatal Fc receptor (FcRn), essential for IgG transport and recycling in dogs and other mammals. FcRn extends the half-life of IgG and albumin, ensuring sustained antibody levels for immune protection by neutralizing pathogens and toxins (53). The SLPI gene encodes a serine protease inhibitor that protects tissues during inflammation by inhibiting enzymes like elastase, with antimicrobial properties contributing to tissue protection during inflammatory responses (Gene Cards).

The Gaddi dog PBMCs indicated few immune-related responses across the treatment groups. In the Control vs. Poly I:C group, the enrichment of the Adaptive Immune System pathway highlights the involvement of key genes like SNAP23, LCP2, ICOS, RNF111, and LRRC41, which are essential for immune responses and T cell activation, suggesting a robust adaptive immune response triggered by Poly I:C (54, 55). The pathways related to Membrane Trafficking and Vesicle-mediated Transport are also significantly represented, involving genes such as STX16, SNAP23, TBC1D15, and NAPG. These pathways are critical for intracellular transport and distribution of proteins and other molecules, which are essential for cellular responses to external stimuli, including immune challenges (56). Moreover, the T Cell Receptor Signaling Pathway is emphasized by the upregulation of NFATC2, PPP2R5A, LCP2, and ICOS, indicating enhanced T cell receptor signaling necessary for T cell activation and differentiation during an immune response (57). Additionally, the Efferocytosis pathway, involving genes like RXRA, TGFBRAP1, ATP2A2, and NFATC2, is also enriched, underscoring its relevance in the immune response (58). The DEGs in the control vs. LPS, group of Gaddi dogs highlight key components of the JAK–STAT signaling pathway and related processes. This pathway is critical for regulating cellular activities such as growth, differentiation, and programmed cell death. The JAK–STAT signaling pathway is notably emphasized in dogs through the KEGG pathway (cfa04630), which underlines its importance in canine cellular communication. According to Kuo et al. (59), lipopolysaccharide (LPS) influences the JAK/STAT pathway by activating Toll-like receptor 4 (TLR4), which then triggers the JAK/STAT cascade. This activation results in elevated production of proinflammatory cytokines, like I6, further highlighting the pathway’s role in mediating inflammation and hypertrophy in cardiac cells. Likewise, CpG treatment has revealed pathways enrichment like The Cytokine-cytokine receptor interaction pathway, featuring genes like CSF3 and IL1R2, which play a vital role in intercellular communication during immune responses (76). The Neurotrophin signaling pathway, involving genes such as MAPK8 and FOXO3, is crucial for neural development and function. Additionally, the FoxO signaling pathway, including genes like FOXO3 and SGK1, is essential for cellular homeostasis and stress resistance (83). A study by Zhang et al. (84) highlights the importance of these pathways in immune responses. For instance, CSF3 is crucial for the proliferation, differentiation, and survival of granulocytes, which are key players in the innate immune response. IL1R2 functions as a decoy receptor, modulating the activity of I1, a major inflammatory mediator. Research indicates that the cytokine-cytokine receptor interaction pathway is fundamental for coordinating the immune system’s response to various stimuli and maintaining immune homeostasis (60).

In the present study, the miRNAome profile of Labrador- and Gaddi dog PBMC was also explored across the three treatment groups and a control group. The miRNA expression analysis in Labrador and Gaddi samples revealed clear differences. Labrador samples had a higher number of expressed miRNAs, predominantly novel. The L-Control group had 2,025 miRNAs (185 known, 1,840 novel), while L-Poly I:C had the most at 2,248 miRNAs (154 known, 2,094 novels).

L-LPS and L-CpG showed 2,068 and 1,946 miRNAs, respectively, with most being novel. In contrast, Gaddi samples had fewer miRNAs, with the G-Control showing 1,259 miRNAs (194 known, 1,065 novel), while G-Poly I:C, G-LPS, and G-CpG had 1,011, 176, and 829 miRNAs, respectively, showing particularly low expression under treatment conditions.

In the analysis of differentially expressed miRNAs across various conditions, 175 miRNAs were common across all treatment groups in Labrador- and Gaddi dogs, with several showing consistent regulation in multiple comparisons. Notably, miR-204, miR-676, miR-106a, miR-335, and miR-132 were DE Gaddi dog PBMCs across in comparisons, indicating their involvement in broad immune responses. Similarly, miR-182, miR-203, and miR-98 were commonly regulated between Control vs. CpG and Control vs. LPS, suggesting their roles in response modulation. miR-196a and miR-144 appeared in comparisons involving Control-Poly I:C, while miR-152 and miR-1 were found across Control-LPS and Control vs. Poly I:C. miR-449a and miR-193b were highlighted in comparisons involving Control vs. CpG, Control vs. CpG, and Control vs. LPS, pointing to their involvement in inflammation. miR-551a and miR-1249 were identified in Control vs. CpG, Control vs. LPS, and Control vs. Poly I:C, reflecting their roles in various stimuli responses. In Gaddi dog PBMCs, miR-582 was uniquely regulated across all treatments. It plays a critical role in early B cell development by inhibiting excessive proliferation of pre-B cells and targeting Hif1α and Rictor involved in mTORC2 signaling (85). For Labrador dog PBMCs, miR-551a and miR-1249 were uniquely expressed. miR-551a functions as an oncogene in head and neck squamous cell carcinoma by targeting GLIPR2 mRNA, which modulates autophagy (61). miR-1249, released by NK cells, induces macrophage apoptosis by targeting anti-apoptotic genes and modulating apoptosis pathways (27, 73).

The differential expression of miR-196a and miR-144 in Labrador PBMCs following Poly I:C stimulation highlights their distinct roles in immune response modulation. miR-196a has been implicated in antiviral defenses and cellular responses to viral infections (86, 87). This suggests that miR-196a could play a role in the early immune response to viral mimics like Poly I:C by enhancing antiviral pathways and bolstering the cell’s defense against viral threats. Meanwhile, miR-144 is involved in the regulation of the NF-κB signaling pathway by targeting and suppressing IκBα expression, as shown in studies by Yang et al. (88) and Zhang et al. (89). By inhibiting IκBα, miR-144 facilitates the activation of p65, a key player in the NF-κB pathway, thereby amplifying NF-κB signaling. This pathway is critical for regulating immune and inflammatory responses, leading to the expression of pro-inflammatory cytokines and other immune-related genes essential for a robust immune response. The findings suggest that miR-144’s enhancement of NF-κB signaling plays a vital role in controlling the inflammatory response triggered by Poly I:C. Together, miR-196a and miR-144 appear to coordinate the regulation of antiviral and inflammatory responses in Labrador-PBMCs—miR-196a by directly managing viral defense and miR-144 by modulating the downstream inflammatory processes for a balanced immune reaction. The research Bamunuarachchi et al. (62) demonstrates that miR-144 impairs the host’s antiviral response by suppressing TRAF6 levels, leading to reduced activation of the IRF7 pathway. *In vivo* studies showed that ablation of miR-144 enhanced resistance to influenza virus infection, indicating its role as a negative regulator of antiviral immunity. Additionally, LPS treatment uniquely regulates cfa-miR-152 and cfa-miR-1. cfa-miR-152 is a tumor suppressor and negatively regulates immune responses by decreasing inflammatory cytokine and type I IFN production in TLR-activated dendritic cells (DCs) (71). It also inhibits the maturation, activation, and function of DCs (80). In contrast, cfa-miR-1 influences critical genes like HSP60, KLF4, and HAND2, which are essential for the physiological functions of smooth and skeletal muscles. This miRNA is also involved in the pathogenesis of various disorders and is dysregulated in several cancers, including gastric, colorectal, breast, prostate, and lung (63, 70, 90).

In Gaddi dog PBMCs, 184 miRNAs were shared across all three treatment groups, while only three miRNAs (cfa-miR-98, 182, and 203) were commonly shared between the Control vs. CpG and Control vs. LPS comparisons. miR-98 regulates SOCS1 and SOCS4, key inhibitors in the JAK–STAT pathway, thus controlling cytokine signaling (75, 91). Targeting SOCS allows JAK–STAT activation, promoting immune cell survival and inflammation, making it relevant in inflammatory disorders (92). miR-182 is primarily linked to cancer and apoptosis, with studies showing it induces apoptosis in rats (93). miR-203 has been associated with combating Canine Atopic Dermatitis (94). In control vs. CpG comparison, three unique miRNAs were identified three unique miRNAs were identified in the Control vs. CpG comparison (cfa-miR-449a, 193b, and 206). miR-449a is involved in immune responses by targeting HDAC1, suppressing IFN-*β* expression, which is crucial for antiviral defense (95). It also regulates cell cycle arrest and apoptosis. miR-206 plays a role in modulating inflammation by regulating neutrophil recruitment in infections and alleviating inflammatory injury via the JAK2/STAT3 pathway, relevant in sepsis and other inflammatory conditions (96, 97).

The unique miRNA in Gaddi dog PBMC across all the treatment groups was cfa-mir-582. A study by (85) showed that miR-582 plays an important role in early B cell development. We have found that miR-582 is essential for inhibiting the excessive proliferation of pre-B cells during B cell development in BM. There is a negative regulator of B-cell proliferation and survival. It achieves this by targeting and downregulating Hif1*α* and Rictor, which are involved in mTORC2 signaling and metabolism. Its downregulation in the Gaddi PBMC indicates the B-cell proliferation against the simulations.

Likewise, two DE miRNAs were unique in Labrador dog PBMC across all the treatment groups including cfa-mir-551a and cfa-mir-1249. In a study Karanam et al. (61), miR-551a is an oncogene in head and neck squamous cell carcinoma (HNSCC). It promotes tumor growth, migration, and invasion. It is associated with poor survival miR-551a directly targets GLIPR2 mRNA, which is a negative regulator of autophagy, and its overexpression leads to reduced GLIPR2 levels, thereby modulating autophagy and affecting the cancer cells’ response to treatment. miR-551a is a microRNA implicated in various biological processes across different animal species. In bovine studies, miR-551a was upregulated in non-pregnant cows’ oviductal epithelial cells, suggesting a role in reproductive physiology (64). In porcine research, miR-551a was highly expressed in milk exosomes, indicating potential involvement in immune system development and fundamental physiological processes (65). These findings suggest that miR-551a plays diverse roles in animal biology, including reproductive functions, immune system regulation, and cancer progression.

miR-1249, found in exosomes released by natural killer (NK) cells and alveolar epithelial cells (AECs), has a role in regulating immune responses and modulating macrophage functions. In the context of NK cells, miR-1249 induces apoptosis in macrophages by targeting anti-apoptotic genes and modulating key components of the apoptosis machinery (66). This results in increased macrophage cell death, contributing to immune regulation. Similarly, miR-1249-5p derived from AECs is transferred to macrophages in response to influenza A virus infection. This exosomal transfer of miR-1249-5p promotes the release of pro-inflammatory cytokines such as TNF-α and IL-6 by inhibiting the expression of solute carrier family four-member 1 (SLC4A1), which activates the NF-κB signaling pathway (74). These dual functions of miR-1249 underscore its role in controlling immune cell interactions, apoptosis, and inflammation, suggesting its potential as a therapeutic target in diseases involving immune dysregulation and infection.

miR-1249, found in exosomes released by natural killer (NK) cells, induces apoptosis in macrophages. When macrophages internalize these NK cell-derived exosomes, the miR-1249 within them can promote cell death. This occurs through several mechanisms: miR-1249 may target and downregulate anti-apoptotic genes, reduce the expression of proteins that prevent apoptosis, or modulate pro-apoptotic pathways by influencing key components of the apoptosis machinery. Additionally, miR-1249 can affect cellular stress responses, such as those related to inflammation and oxidative stress, further contributing to macrophage apoptosis. This process highlights miR-1249’s role in regulating immune cell interactions and its potential as a target for therapeutic strategies (53). miR-1249-5p, derived from alveolar epithelial cells (AECs), plays a role in immune regulation during lung infections. In response to influenza A virus infection, miR-1249-5p is transferred via exosomes from lung epithelial cells to macrophages. This transfer promotes the release of pro-inflammatory cytokines TNF-α and IL-6 by suppressing the expression of solute carrier family four member 1 (SLC4A1), activating the NF-κB signaling pathway (67). The unique miRNAs, miR-551a and miR-1249, in the Gaddi dog breed, offer a valuable opportunity to explore their regulatory roles in gene expression, specifically in dogs. Studying these miRNAs could reveal insights into breed-specific adaptations, immune responses, and resistance to environmental stressors or diseases.

Insights on differential systems biology on Labrador home-owned and exotic origin contrarily Gaddi dogs, reared by locals of the sub-Himalayan region to guard and herd livestock, and are free-range dogs. Hence, the evolution of the immunological systems must have some differences. The mRNA-Seq analysis of Labrador PBMCs revealed consistent enrichment of Th1, Th2, and Th17 differentiation pathways across all treatment groups. In contrast, Gaddi dog PBMCs also showed enrichment in Th1 and Th17 differentiation pathways and inflammatory pathways, chemokine and cytokine signaling, apoptosis, cell proliferation, and T-cell activation pathways across the treatment groups. These findings suggest a heightened immune response in Gaddi dogs against various TLR ligands. The miRNA-Seq analysis identified a significant overlap in miRNA Between the two breeds, predominantly regulating transcription factors, the cell cycle, autophagy, TLR signaling, and cytokine and chemokine pathways. However, breed-specific miRNAs were observed: Labradors exhibited cfa-miR-196a, miR-144, miR-204, miR-676, miR-106a, miR-335, and miR-132 across treatment groups, whereas Gaddi dogs showed cfa-miR-449a, miR-193b, miR-206, miR-551a, and miR-1249. Moreover, a study by Guo et al. (68) explored the role of miR-1249 in intervertebral disc degeneration (IDD) and being used as a target treatment for IDD. These findings underscore the distinct transcriptomic and miRNAomic landscapes between the two genetics groups, reflecting their diverse immunogenetic adaptations and potential disease resistance mechanisms based on pathway analysis.

## 5 Conclusion

We present the inaugural atlas of mRNA and miRNA expression in TLR ligand-stimulated PBMCs of Labrador (exotic) and Gaddi (Indigenous) dogs. This reference will support future functional studies and challenge experiments to identify disease-associated genes and immune pathways. Additionally, it provides a maiden report on differential transcriptomes, contributing to breed recognition of the Gaddi dog.

## Data Availability

The datasets presented in this study can be found in online repositories. The names of the repository/repositories and accession number(s) can be found at: https://www.ncbi.nlm.nih.gov/, PRJNA1035251.

## Footnotes

^1^ https://ngdc.cncb.ac.cn/databasecommons/database/id/167

^2^ david.ncifcrf.gov/

^3^ www.genome.jp/kegg/pathway.html

^4^ reactome.org/userguide/pathway-browser

^5^ http://pantherdb.org/

^6^ https://string-db.org/

^7^ www.bioinformatics.com.cn/en

^8^ www.targetscan.org/vert_80

^9^ mirdb.org/

^10^ mirtarbase.cuhk.edu .cn
