# Supplementary material for: In-vitro transcriptomic profiling of indigenous Gaddi vis-à-vis exotic Labrador dogs: insights from systems biology
Source: Front Vet Sci. 2025 Jun 20;12:1489905. doi: 10.3389/fvets.2025.1489905 (PMC12226298; doi:10.3389/fvets.2025.1489905)
Supplement: Supplementary file 1 [file Data_Sheet_1.docx]

***In-Vitro* Transcriptomic Profiling of Indigenous Gaddi vis-à-vis Exotic Labrador Dogs: Insights from Systems Biology**

**Jaswinder Kaur**^1^**, Manu. M^2^, Bilawal Singh^3^, R.S Sethi^4^, Deepti Narang^5^, Simarjeet Kaur^6^, CS Mukhopadhyay^*^**

^1^Ph.D. Scholar, ^2^Assistant Professor, ^3^Assistant Professor, ^4^Additional Director of Research, ^5^Principal Scientist, ^6^Principal Scientist, ^*^Senior Scientist,

^2^ Department of Microbial and Environmental Biotechnology College of Animal Biotechnology, ^3^ Department of Veterinary Gynecology & Obstetrics, ^4^Additional Director of Research Cum Dean at the College of Dairy Science and Food Technology, ^5^Department of Veterinary Microbiology, College of Veterinary Sciences, ^6^Department of Animal Genetics and Breeding, College of Veterinary Sciences ^1*^ Department of Bioinformatics, College of Animal Biotechnology,

Guru Angad Dev Veterinary and Animal Science University, Ludhiana, Punjab, India,

*Email: [csmukhopadhyay@gadvasu.in](mailto:csmukhopadhyay@gadvasu.in)

**Supplementary Figures**


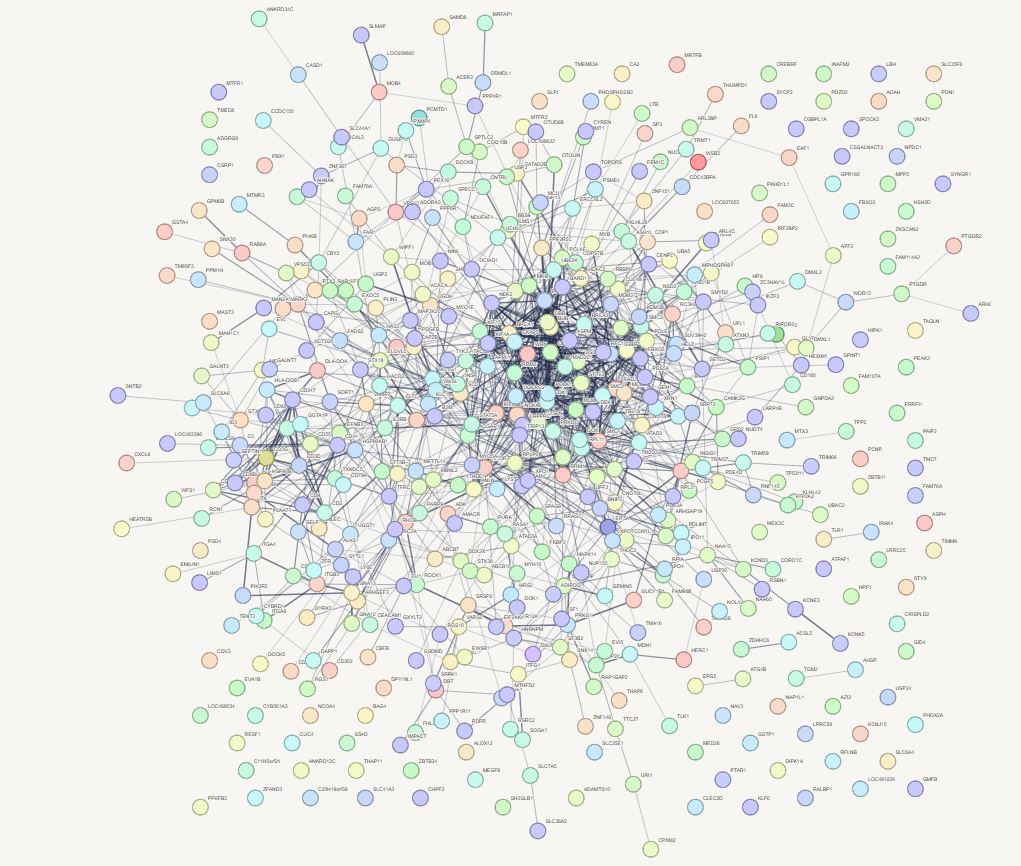


**A**


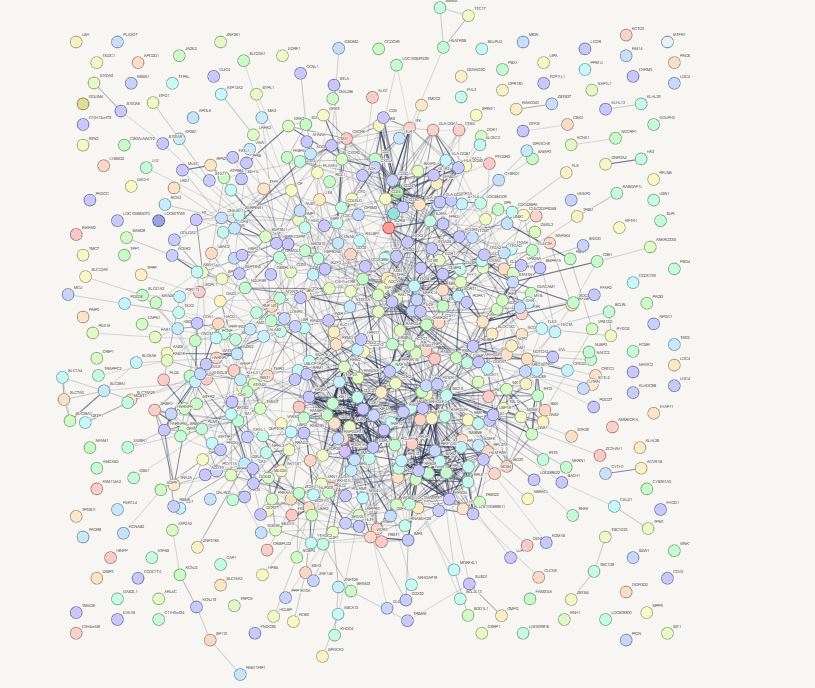


**B**


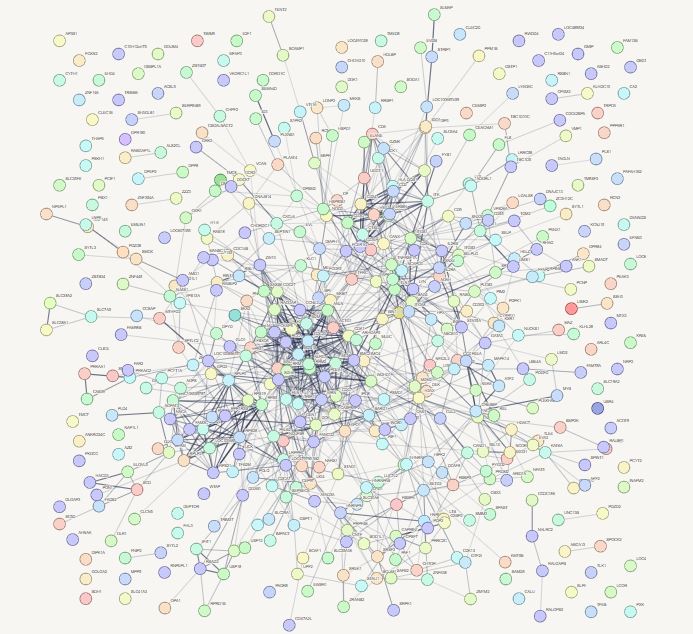


**C**

**Supplementary Figure 1: PPI Network Analysis using STRING tool** (<https://string-db.org/>) **of Dysregulated Genes in Labrador PBMCs A. (Ctrl- LPS), B. Ctrl-LPS, and C. Ctrl-CpG**


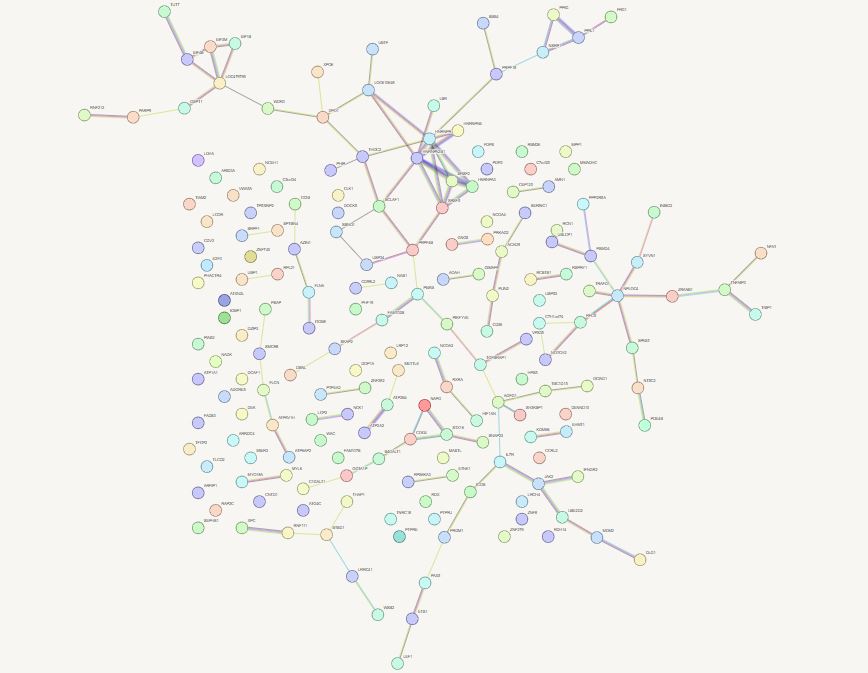


**A**


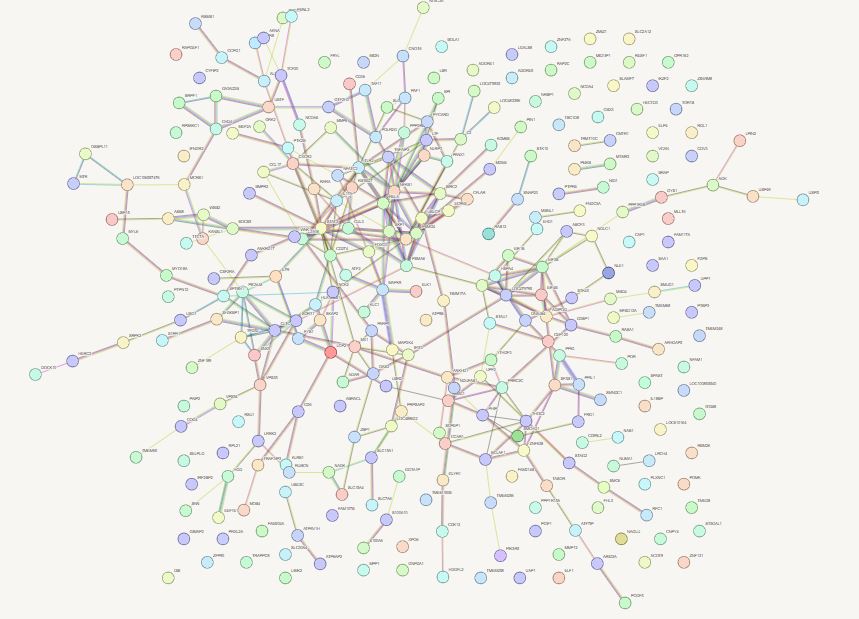


**B**


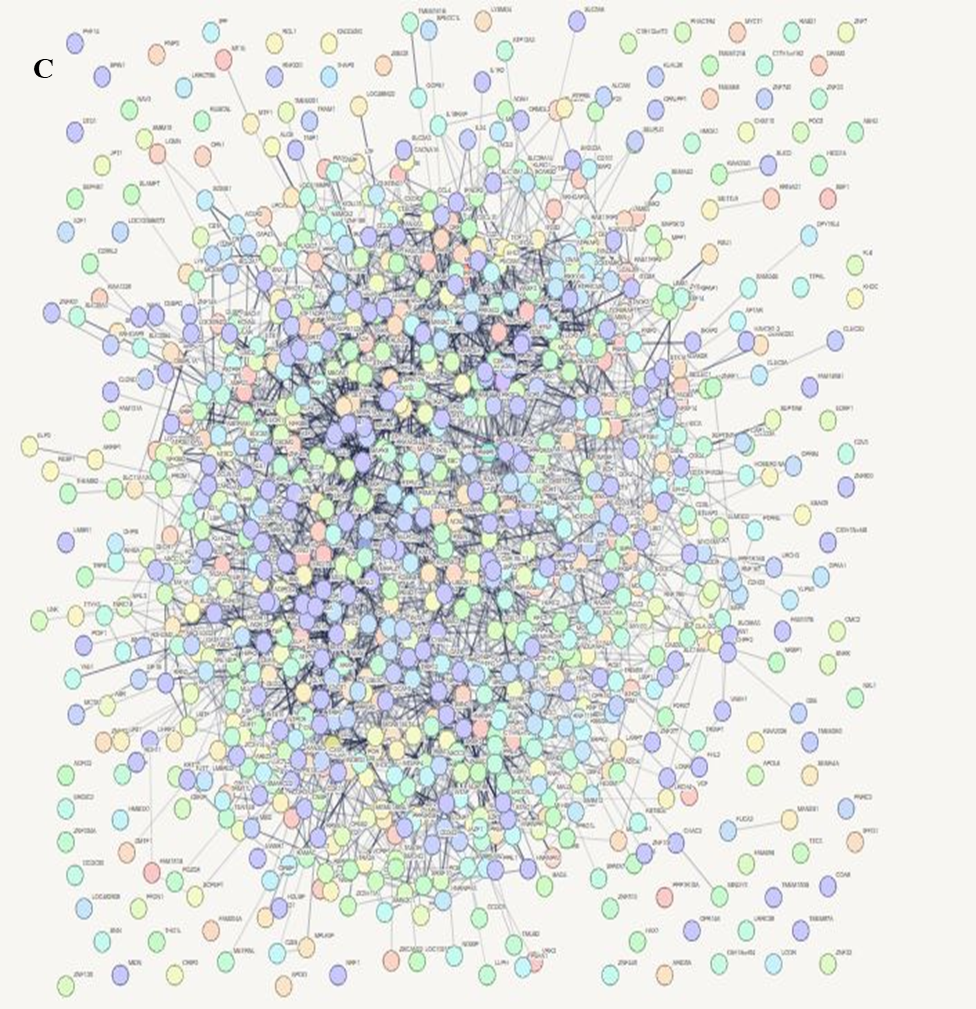


**Supplementary Figure 2: PPI Network Analysis using STRING tool** (<https://string-db.org/>) **of Dysregulated Genes in *Gaddi* PBMCs A. (Ctrl- LPS), B. Ctrl-LPS, and C. Ctrl-CpG**


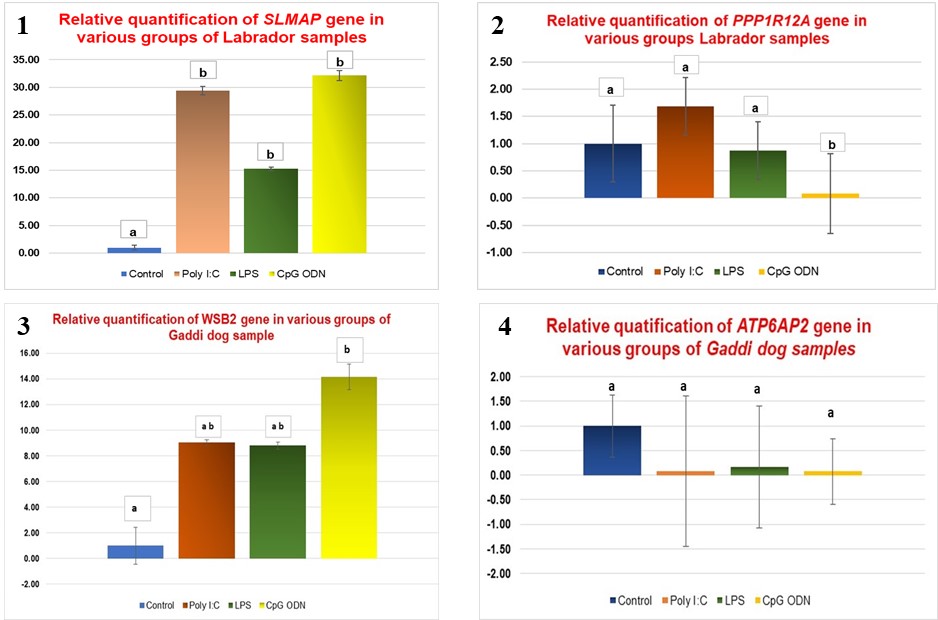


**Supplementary Figure 2: Relative expression of mRNA Genes in Labrador and *Gaddi* dog PBMCs common across all the groups. 1.PPP1R12A was significantly (p<0.05) downregulated, 2. SLMAP was significantly (p<0.05) upregulated, 3. WSB2 was significantly (p<0.05) upregulated, 4. ATP6AP2 was downregulated (p>0.05).** a, b: No common superscript between the levels of the effect indicates a significant difference at p<0.05


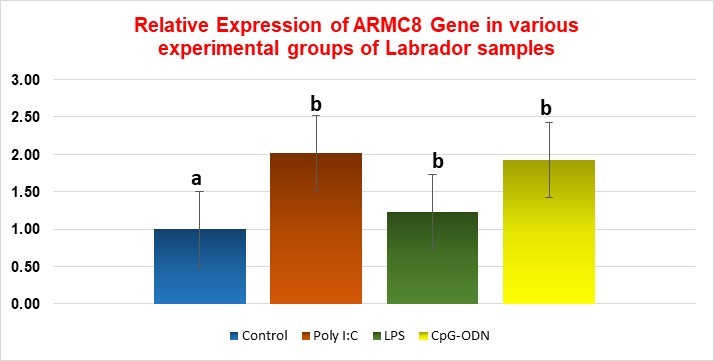

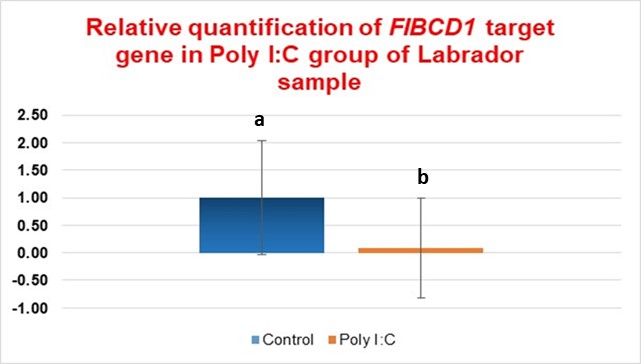

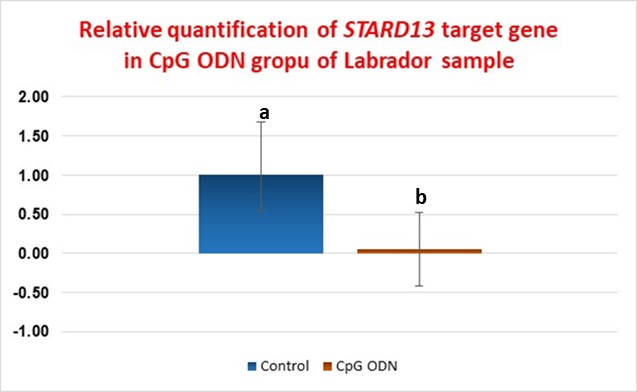

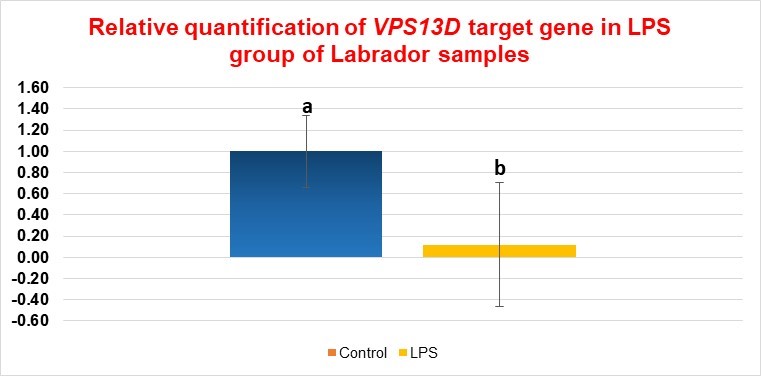


**1**

**2**

**3**

**4TLR 4 Ligand, (LPS) @ 100ng/ml for 6hrs**

**Supplementary Figure 3: Relative expression of miRNA target Genes in Labrador dog PBMC common in various treatment groups. 1. *ARMC8* is significantly (p<0.05) upregulated in all the treatment groups, 2. *FIBCD1* was significantly (p<0.05) downregulated in ctrl vs PolyIC group, 3. *STARD13* was significantly (p<0.05) downregulated ctrl vs CpG group, 4. *VPS13D* was significantly (p<0.05) downregulated in the ctrl vs LPS group.** a, b: No common superscript between the levels of the effect indicates a significant difference at p<0.05


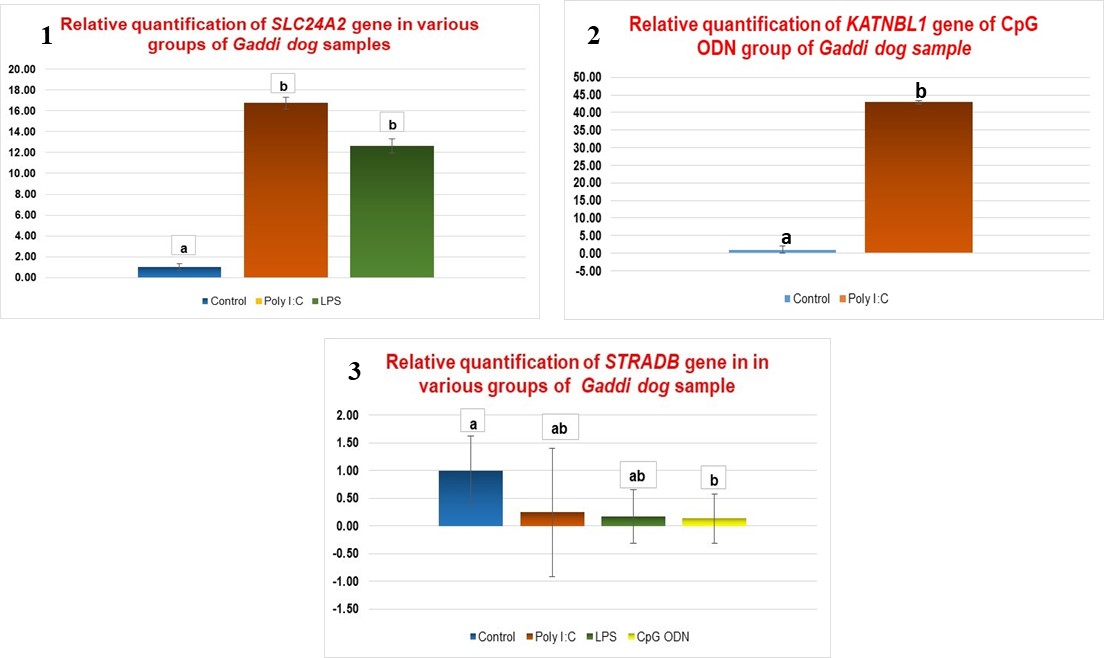


**Supplementary Figure 4: Relative expression of miRNA target Genes in *Gaddi* dog PBMC common in various treatment groups. 1. SLC24A2 was significantly (p<0.05) upregulated in ctrl vs Poly IC and LPS groups, 2. KATNBL1 was significantly (p<0.05) upregulated in the ctrl vs CpG group, 3. *STARD13* was significantly (p<0.05) downregulated ctrl vs CpG group, 4. STRADB was significantly (p<0.05) downregulated in all the experimental groups.** a, b: No common superscript between the levels of the effect indicates a significant difference at

**Supplementary Tables**

**Supplementary table 1: Details of Dogs for Sample Collection (Labrador and *Gaddi* Dogs)**

| **S. No.** | **Location of sample collection** | **Name/ID of dog** | **Age/Sex** | **Health status** | **TLC & DLC report** |
| --- | --- | --- | --- | --- | --- |
| 1 | Dog House | Dog no. 3 | 5 years / Male | Apparently Healthy | No Infection |
| 2 | Dog House | Dog no. 6 | 5.5 years / Male | Apparently Healthy | No Infection |
| 3 | Panchrukhi Vill. Palampur, Kangra, H.P | Shera, Baan *Gaddi* | 4.5 yr/ Male | Apparently Healthy | No Infection |
| 4 | Kennel, Barnala, Punjab | Tiger, *Gaddi* dog | 6 yrs/ Male | Apparently Healthy | No Infection |

**Supplementary table 2: Details of Blood samples TLC and DLC for Labrador dogs and *Gaddi* dogs**

| **Parameters** | **Units** | **Labrador dogs** | | ***Gaddi* Dogs** | |
| --- | --- | --- | --- | --- | --- |
|  |  | **Dog No.3** | **Dog No.6** | **Shera dog** | **Tiger dog** |
| Total Leukocyte count | cu mm | 8700 | 7890 | 8750 | 9690 |
| Differential Leukocyte count | | | | | |
| Neutrophiles | % | 43 | 45 | 53 | 63 |
| Lymphocytes | % | 11 | 8 | 7 | 12 |
| Monocytes | % | 3 | 7 | 4 | 8 |
| Basophils | % | - | - | - | 2 |
| Eosinophils | % | - | - | - | - |
| Hemoglobin (Hbg) | g/dl | 13.8 | 14.2 | 13.7 | 12.8 |

**Supplementary table 3: mRNA Seq Genes selected for validation with their Primer Sequences and Endogenous controls**

| **Gene name** | **Breed/ Experimental group** | **Dysregulation** | **Primer** | **Sequence (5’-3’)** | **Primer length** | **GC%** | **Tm** |
| --- | --- | --- | --- | --- | --- | --- | --- |
| ***PPP1R12A***  ***(***Protein Phosphatase 1 Regulatory Subunit 12A***)*** | Labrador Dog Across all the treatment group | Downregulated | **F' Primer** | AGTGTTCCAAGCACCACATC | **20** | 50 | 58.39 |
|  |  |  | **R' Primer** | AGTGAGGTATGATCTGCTTTGT | **22** | 40.91 | 57.17 |
| ***SLMAP***  ***(***Sarcolemma-Associated Protein***)*** |  | Upregulated | **F' Primer** | ACGGGCAAGTTTTATCTCCA | **20** | 45 | 57.13 |
|  |  |  | **R' Primer** | TTCCGTGTGTTCTCCGTTAC | **20** | 50 | 57.86 |
|  | *Gaddi* Dog Across all the treatment group | Upregulated | **F' Primer** | AAGCTAGAGGGCCACCAAAG | **20** | 55 | 59.67 |
| ***WSB2***  ***(***(WD Repeat and SOCS Box Containing 2***)*** |  |  | **R' Primer** | GTTGCGTGTGGTGGAGTAAC | **20** | 55 | 59.42 |
|  |  | Downregulated | **F' Primer** | CCTTTATGGCGGGAATGCAG | **20** | 55 | 59.33 |
| ***ATP6AP2***  ***(***ATPase H+ Transporting Accessory Protein 2***)*** |  |  | **R' Primer** | GGCTTGATGGGTTCTTCGCT | **20** | 55 | 60.68 |
| ***GAPDH*** | Endogenous Control | | **F’ Primer** | GAGATCCCGCCAACATCAAATGG | **23** | 52.17 | 62.12 |
|  |  |  | **R’ Primer** | GAGATCCCGCCAACATCAAATGG | **23** | 47.83 | 61.95 |
| ***Beta Actin*** |  |  | **F’ Primer** | TTCGAGACTTTCAACACCCC | **20** | 50 | 57.75 |
|  |  |  | **R’ Primer** | CACCGGAGTCCATCACGA | **18** | 55 | 57.72 |

**Supplementary table 4: Target genes with their primer sequences of miRNAs from PBMCs of Labrador breed PBMC across all the treatment groups selected for validation**

| **Gene name** | **Experimental Group** | **Dysregulation** | **Primer** | **Sequence (5’-3’)** | **Primer length** | **GC%** | **Tm** |
| --- | --- | --- | --- | --- | --- | --- | --- |
| ***ARMC8***  ***(***Armadillo Repeat Containing 8***)*** | Across all the experimental groups | **Upregulated** | **F' Primer** | ATAGCAGATGGGACAACAGC | **20** | 50 | 57.66 |
|  |  |  | **R' Primer** | ATGCCCATGTCCCGTAATT | **20** | 45 | 57.55 |
| ***FIBCD1***  (Fibrinogen C Domain Containing 1) | Control vs PolyIC | **Downregulated** | **F' Primer** | GACCACTCGGAGAACAACTG | **20** | 55 | 57.97 |
|  |  |  | **R' Primer** | TCCGAGAACTTGAGCGAGTA | **20** | 50 | 58.22 |
| ***VPS13D***  ***(***Vacuolar Protein Sorting 13D***)*** | Control vs LPS | **Downregulated** | **F' Primer** | AGGAAAGGCACAAAGTGGTT | **20** | 45 | 58.18 |
|  |  |  | **R' Primer** | GACTAGAAACGACCGGGTGA | **20** | 43 | 57.84 |
| ***STARD13***  ***(***StAR Related Lipid Transfer Domain Containing 13***)*** | Control vs CpG | **Downregulated** | **F' Primer** | AGAGCGAGCAGAGTCAGTAT | **20** | 50 | 57.97 |
|  |  |  | **R' Primer** | ACTACCTCTTGGAGCCCATT | **20** | 50 | 58.03 |

**Supplementary table 5: Target genes with their primer sequences of miRNAs from PBMCs of Gaddi dog across all the treatment groups selected for validation**

| **Gene name** | **Experimental Group** | **Dysregulation** | **Primer** | **Sequence (5’-3’)** | **Primer length** | **GC%** | **Tm** |
| --- | --- | --- | --- | --- | --- | --- | --- |
| **SLC24A2**  **(**Solute Carrier Family 24 Member 2**)** | Control vs Poly IC and LPS | **Upregulated** | **F' Primer** | TCCCTCCACAACAGTCTCAT | **20** | 50 | 57.97 |
|  |  |  | **R' Primer** | CCTCCCTTCTTCAGTCATTGT | **21** | 47.62 | 57.29 |
| **KATNBL1**  **(**Katanin Regulatory Subunit B1 Like 1**)** | Control vs CpG | **Upregulated** | **F' Primer** | AAAAGGTGGTGGTCAGAACT | **20** | 45 | 56.88 |
|  |  |  | **R' Primer** | GCGTCTACATCCTTGGCTAT | **20** | 50 | 57.18 |
| **STRADB**  **(**StarDB**)** | Across all the treatment groups | **Down-regulated** | **F' Primer** | CCCACCTGCATAGTTTGGTT | **20** | 50 | 58.08 |
|  |  |  | **R' Primer** | CACGCTGTAATCCCAACACT | **20** | 50 | 58.19 |

**Supplementary table 6: Functional classification of upregulated genes of Ctrl vs Poly IC Labrador dog according to the pathways they influence and control**

| **S. No.** | **Term** | **Count** | **Genes** |
| --- | --- | --- | --- |
| 1 | cfa04640: Hematopoietic cell lineage | 11 | CD2, DLA-DOA, CD8B, LOC100856137, ITGB3, CD9, CD3G, CD3E, CD3D, CD55, CD44 |
| 2 | cfa05166: Human T-cell leukemia virus 1 infection | 9 | STAT5A, CREBBP, DLA-DOA, LOC100856137, IL2RB, CD3G, CD3E, CD3D, B2M |
| 3 | cfa04658: Th1 and Th2 cell differentiation | 8 | STAT5A, DLA-DOA, LOC100856137, IL2RB, CD3G, CD247, CD3E, CD3D |
| 4 | cfa04659: Th17 cell differentiation | 8 | STAT5A, DLA-DOA, LOC100856137, IL2RB, CD3G, CD247, CD3E, CD3D |
| 5 | cfa05169: Epstein-Barr virus infection | 8 | DLA-DOA, LOC100856137, CD3G, CD247, CD3E, CD3D, B2M, CD44 |
| 6 | cfa04660: T cell receptor signaling pathway | 7 | ITK, CD8B, CD3G, PPP2R5C, CD247, CD3E, CD3D |
| 7 | cfa04514: Cell adhesion molecules | 6 | CD2, DLA-DOA, CD8B, LOC100856137, CD6, ITGA8 |
| 8 | cfa04820: Cytoskeleton in muscle cells | 6 | CAPZB, CSRP1, ITGB3, ITGA8, FHL3, ACTG1 |
| 9 | cfa04144: Endocytosis | 6 | SH3GLB1, EHD1, CAPZB, PSD4, IL2RB, PSD3 |
| 10 | cfa04810: Regulation of actin cytoskeleton | 6 | LOC102156243, LOC102152706, ITGB3, ITGA8, NCKAP1L, ACTG1 |
| 11 | cfa05162: Measles | 5 | STAT5A, IL2RB, CD3G, CD3E, CD3D |
| 12 | cfa04145: Phagosome | 5 | DLA-DOA, LOC100856137, ITGB3, STX18, ACTG1 |
| 13 | cfa04613: Neutrophil extracellular trap formation | 5 | H4C8, GSDMD, H2AX, ITGB3, ACTG1 |
| 14 | cfa05340: Primary immunodeficiency | 4 | CD79A, CD8B, CD3E, CD3D |
| 15 | cfa04612: Antigen processing and presentation | 4 | DLA-DOA, CD8B, LOC100856137, B2M |
| 16 | cfa05416: Viral myocarditis | 4 | DLA-DOA, LOC100856137, CD55, ACTG1 |
| 17 | cfa05323: Rheumatoid arthritis | 4 | DLA-DOA, LOC100856137, LOC106557449, LTB |
| 18 | cfa05235: PD-L1 expression and PD-1 checkpoint pathway in cancer | 4 | CD3G, CD247, CD3E, CD3D |
| 19 | cfa05142: Chagas disease | 4 | CD3G, CD247, CD3E, CD3D |

**Supplementary table 8: Functional classification of Down-regulated genes of Ctrl vs Poly IC Labrador dog according to the pathways they influence and control**

| **S. No.** | **Term** | **Count** | **Genes** |
| --- | --- | --- | --- |
| 1 | cfa01100: Metabolic pathways | 41 | SUV39H2, SETD2, GUCY1B1, ALOX15, ADK, HACD3, HACD2, GGTA1, ACACA, FADS2, UGP2, SPTLC2, MAN2A1, CA2, NSD2, DBT, MAN1C1, BPNT2, ST3GAL5, GALNT7, RRM1, RRM2, GALNT3, ELOVL5, PDE4D, ACSL5, ASH1L, DCK, RPIA, UGDH, PHOSPHO2, AMACR, GNPDA2, GSTA4, MTHFD2, SCD, AGPS, PDE3A, STT3B, GCLM, IDO1 |
| 2 | cfa04110: Cell cycle | 14 | HDAC2, PRKDC, PLK1, SMC3, PDS5A, ORC5, CDC20, MCM3, MDM2, CDK1, MCM6, TRIP13, FBXO5, BUB1 |
| 3 | cfa04810: Regulation of actin cytoskeleton | 10 | GNA13, DIAPH2, PPP1R12A, ROCK1, ITGA4, GNA12, PDGFB, WASL, SSH3, MYH10 |
| 4 | cfa04022: cGMP-PKG signaling pathway | 9 | GNA13, PPP1R12A, GUCY1B1, ROCK1, INSR, GNA12, PDE3A, ATP2A2, PRKG1 |
| 5 | cfa04120: Ubiquitin mediated proteolysis | 9 | CDC20, COP1, UBB, HERC1, MDM2, TRIM37, BRCA1, UBE2K, CUL4B |
| 6 | cfa04114: Oocyte meiosis | 8 | CDC20, PLK1, CDK1, FBXO5, SMC3, MAPK14, BUB1, CAMK2G |
| 7 | cfa01212: Fatty acid metabolism | 7 | FADS2, ELOVL5, SCD, ACSL5, HACD3, HACD2, ACACA |
| 8 | cfa04270: Vascular smooth muscle contraction | 7 | GNA13, PPP1R12A, GUCY1B1, ROCK1, GNA12, MYH10, PRKG1 |
| 9 | cfa00230: Purine metabolism | 7 | RRM1, RRM2, GUCY1B1, PDE4D, ADK, PDE3A, DCK |
| 10 | cfa04146: Peroxisome | 6 | AMACR, AGPS, PXMP4, ACSL5, FAR1, CROT |
| 11 | cfa03013: Nucleocytoplasmic transport | 6 | IPO11, XPO1, NUP155, XPO4, XPOT, THOC2 |
| 12 | cfa04611: Platelet activation | 6 | GNA13, PPP1R12A, GUCY1B1, ROCK1, MAPK14, PRKG1 |
| 13 | cfa01040: Biosynthesis of unsaturated fatty acids | 5 | FADS2, ELOVL5, SCD, HACD3, HACD2 |
| 14 | cfa04216: Ferroptosis | 5 | TFRC, NCOA4, ALOX15, ACSL5, GCLM |
| 15 | cfa03030: DNA replication | 4 | RFC1, MCM3, MCM6, POLE |
| 16 | cfa03440: Homologous recombination | 4 | BARD1, RBBP8, BRCA1, BRCA2 |
| 17 | cfa00062: Fatty acid elongation | 3 | ELOVL5, HACD3, HACD2 |

**Supplementary table 8: Functional classification of Upregulated genes of Ctrl vs LPS Labrador dog according to the pathways they influence and control**

| **S. No.** | **Term** | **Count** | **Genes** |
| --- | --- | --- | --- |
| 1 | cfa05200: Pathways in cancer | 15 | ITGB1, STAT5A, RALBP1, CREBBP, EGF, GSTP1, NCOA3, ITGA2, ITGA2B, AXIN1, CDH1, IL2RB, GNAS, ARHGEF1, RAC1 |
| 2 | cfa04640: Hematopoietic cell lineage | 13 | DLA-DOA, ITGB3, ITGA2, ITGA2B, CD3G, CD3E, CD3D, CD2, CD8B, LOC100856137, CD9, DLA-DRA, CD55 |
| 3 | cfa04810: Regulation of actin cytoskeleton | 13 | ITGB1, CYFIP2, LOC102156243, EGF, LOC102156038, ITGB3, ITGA2, ITGA2B, ACTG1, LOC102152706, ITGA8, ARHGEF1, RAC1 |
| 4 | cfa03010: Ribosome | 13 | LOC119863879, RPLP0, LOC119864697, LOC100685611, RPL6, RPS15, RPS28, RPS19, RPL37A, RPL36, RPLP2, RPS21, RPS24 |
| 5 | cfa05171: Coronavirus disease - COVID-19 | 13 | LOC119863879, RPLP0, LOC119864697, LOC100685611, RPL6, RPS15, RPS28, RPS19, RPL37A, RPL36, RPLP2, RPS21, RPS24 |
| 6 | cfa05166: Human T-cell leukemia virus 1 infection | 12 | STAT5A, CREBBP, DLA-DOA, ATF6B, LOC100856137, IL2RB, NFATC2, CD3G, CD3E, CD3D, B2M, DLA-DRA |
| 7 | cfa04658: Th1 and Th2 cell differentiation | 11 | STAT5A, DLA-DOA, LOC100856137, IL2RB, NFATC2, CD3G, GATA3, CD247, CD3E, CD3D, DLA-DRA |
| 8 | cfa04659: Th17 cell differentiation | 11 | STAT5A, DLA-DOA, LOC100856137, IL2RB, NFATC2, CD3G, GATA3, CD247, CD3E, CD3D, DLA-DRA |
| 9 | cfa05169: Epstein-Barr virus infection | 11 | PSMD12, DLA-DOA, LOC100856137, TAP1, CD3G, RAC1, CD247, CD3E, CD3D, B2M, DLA-DRA |
| 10 | cfa04015: Rap1 signaling pathway | 11 | ITGB1, RAP1B, CDH1, EGF, ITGB3, ITGA2B, GNAS, EVL, RAC1, RAPGEF5, ACTG1 |
| 11 | cfa04151: PI3K-Akt signaling pathway | 11 | ITGB1, GHR, PRKAA1, ATF6B, EGF, ITGB3, ITGA2, IL2RB, ITGA2B, ITGA8, RAC1 |
| 12 | cfa04514: Cell adhesion molecules | 10 | CD2, ITGB1, DLA-DOA, CD40LG, CD8B, LOC100856137, CD6, CDH1, ITGA8, DLA-DRA |
| 13 | cfa04660: T cell receptor signaling pathway | 9 | ITK, CD40LG, CD8B, NCK2, NFATC2, CD3G, CD247, CD3E, CD3D |
| 14 | cfa04611: Platelet activation | 9 | ITGB1, RAP1B, ITGB3, ITGA2, ITGA2B, GNAS, ARHGEF1, GP6, ACTG1 |
| 15 | cfa04145: Phagosome | 9 | ITGB1, DLA-DOA, LOC100856137, ITGB3, ITGA2, TAP1, RAC1, DLA-DRA, ACTG1 |
| 16 | cfa04510: Focal adhesion | 9 | ITGB1, RAP1B, EGF, ITGB3, ITGA2, ITGA2B, ITGA8, RAC1, ACTG1 |
| 17 | cfa04144: Endocytosis | 9 | SH3GLB1, GRK3, GRK5, BIN1, PSD4, IL2RB, ARAP1, CYTH1, EPN1 |

**Supplementary table 9: Functional classification of Down-regulated genes of Ctrl vs LPS Labrador dog according to the pathways they influence and control**

| **S. No.** | **Term** | **Count** | **Genes** |
| --- | --- | --- | --- |
| 1 | cfa05165: Human papillomavirus infection | 12 | PTGER4, NOTCH2, PPP2R1B, CCNE2, MX1, CDK2, UBR4, ISG15, ITGB7, PSEN1, PTGS2, JAK1 |
| 2 | cfa05166: Human T-cell leukemia virus 1 infection | 11 | CDC20, EGR1, CRTC3, CCNE2, ESPL1, IL1R2, IL2RA, CDK2, E2F2, ADCY6, JAK1 |
| 3 | cfa04148: Efferocytosis | 10 | PTGER4, CEBPB, RAB5C, ALOX15, TGFBRAP1, ATP2A2, MAPK14, PTGS2, CAMK2G, PBX1 |
| 4 | cfa04110: Cell cycle | 10 | CDC20, DBF4, PPP2R1B, CCNE2, ESPL1, CDK2, CDK1, E2F2, CDC7, KNL1 |
| 5 | cfa05169: Epstein-Barr virus infection | 10 | OAS1, CCNE2, OAS2, OAS3, CDK2, E2F2, ISG15, MAPK14, LOC490269, JAK1 |
| 6 | cfa04144: Endocytosis | 10 | RAB11FIP1, RAB5C, RAB31, TFRC, WIPF1, IL2RA, CXCR2, VPS26A, ARAP1, CYTH1 |
| 7 | cfa04114: Oocyte meiosis | 9 | CDC20, PPP2R1B, CCNE2, ESPL1, CDK2, CDK1, MAPK14, CAMK2G, ADCY6 |
| 8 | cfa04621: NOD-like receptor signaling pathway | 9 | HSP90AB1, CXCL8, OAS1, OAS2, OAS3, ERBIN, MAPK14, MCU, JAK1 |
| 9 | cfa05152: Tuberculosis | 9 | CEBPB, RAB5C, ITGAM, LOC611538, FCGR1A, MAPK14, CAMK2G, HSPD1, JAK1 |
| 10 | cfa05162: Measles | 8 | OAS1, CCNE2, OAS2, OAS3, IL2RA, MX1, CDK2, JAK1 |
| 11 | cfa04218: Cellular senescence | 8 | CXCL8, CCNE2, CDK2, CDK1, E2F2, CAPN1, MAPK14, MCU |
| 12 | cfa05160: Hepatitis C | 8 | OAS1, PPP2R1B, OAS2, OAS3, MX1, CDK2, E2F2, JAK1 |
| 13 | cfa04657:IL-17 signaling pathway | 7 | CEBPB, HSP90AB1, CXCL8, MAPK14, PTGS2, S100A9, HSP90B1 |
| 14 | cfa04640: Hematopoietic cell lineage | 7 | CSF3R, ITGAM, TFRC, IL1R2, IL2RA, FCGR1A, LOC490269 |

**Table 10: Functional classification of Upregulated genes of Ctrl vs CpG Labrador dog according to the pathways they influence and control**

| **S. No.** | **Term** | **Count** | **Genes** |
| --- | --- | --- | --- |
| 1 | cfa05171: Coronavirus disease - COVID-19 | 16 | LOC119863879, RPS5, RPLP0, LOC607833, LOC119864483, LOC100685611, RELA, LOC106559375, TNFRSF1A, RPS28, RPS19, LOC106559787, RPL37A, RPLP2, RPS21, RPS24 |
| 2 | cfa03010: Ribosome | 14 | LOC119863879, RPS5, RPLP0, LOC607833, LOC119864483, LOC100685611, LOC106559375, RPS28, RPS19, LOC106559787, RPL37A, RPLP2, RPS21, RPS24 |
| 3 | cfa05200: Pathways in cancer | 11 | ITGB1, STAT5A, RALBP1, CREBBP, EGF, GSTP1, NCOA3, IL2RB, PIM2, RASGRP2, RELA |
| 4 | cfa04640: Hematopoietic cell lineage | 10 | CD2, CD8B, LOC100856137, ITGB3, CD9, CD3G, CD3E, CD3D, CD55, LOC490269 |
| 5 | cfa05166: Human T-cell leukemia virus 1 infection | 10 | STAT5A, CREBBP, LOC100856137, IL2RB, CD3G, CD3E, CD3D, B2M, RELA, TNFRSF1A |
| 6 | cfa04658: Th1 and Th2 cell differentiation | 9 | STAT5A, LOC100856137, IL2RB, CD3G, GATA3, CD247, CD3E, CD3D, RELA |
| 7 | cfa04659: Th17 cell differentiation | 9 | STAT5A, LOC100856137, IL2RB, CD3G, GATA3, CD247, CD3E, CD3D, RELA |
| 8 | cfa05169: Epstein-Barr virus infection | 8 | LOC100856137, CD3G, CD247, CD3E, CD3D, B2M, RELA, LOC490269 |

**Supplementary table 11: Functional classification of downregulated genes of Ctrl vs CpG Labrador dog according to the pathways they influence and control**

| **S. No.** | **Term** | **Count** | **Genes** |
| --- | --- | --- | --- |
| 1 | cfa04110: Cell cycle | 14 | BUB1B, CDC7, CDC14B, CDC20, STAG1, RBL1, CCNE2, CDC27, E2F1, MDM2, CDK1, MCM6, TRIP13, FBXO5 |
| 2 | cfa05417: Lipid and atherosclerosis | 10 | LYN, SELP, PLCB3, PDPK1, CASP3, OLR1, MAPK14, HSPD1, HSP90B1, NFE2L2 |
| 3 | cfa05166: Human T-cell leukemia virus 1 infection | 10 | CDC20, ATF2, XPO1, CCNE2, IL2RA, CANX, CDC27, E2F1, BUB1B, TLN1 |
| 4 | cfa05203: Viral carcinogenesis | 9 | LYN, CDC20, ATF2, RBL1, CCNE2, CASP3, UBR4, MDM2, CDK1 |
| 5 | cfa05169: Epstein-Barr virus infection | 8 | LYN, CCNE2, CASP3, E2F1, MDM2, PSMD1, MAPK14, LOC490269 |
| 6 | cfa04218: Cellular senescence | 7 | RBL1, CCNE2, SERPINE1, E2F1, MDM2, CDK1, MAPK14 |
| 7 | cfa04120: Ubiquitin-mediated proteolysis | 7 | CDC20, UBE2D2, CUL2, CDC27, MDM2, UBE4A, TRIM37 |
| 8 | cfa05202: Transcriptional misregulation in cancer | 7 | NCOR1, EWSR1, NSD2, MDM2, BMP2K, ELANE, PBX1 |
| 9 | cfa04115: p53 signaling pathway | 6 | RRM2, CCNE2, CASP3, SERPINE1, MDM2, CDK1 |
| 10 | cfa04114: Oocyte meiosis | 6 | CDC20, CCNE2, CDC27, CDK1, FBXO5, MAPK14 |
| 11 | cfa01212: Fatty acid metabolism | 5 | FADS2, ELOVL5, SCD, ACSL5, HACD3 |
| 12 | cfa03320: PPAR signaling pathway | 5 | FADS2, SCD, PDPK1, OLR1, ACSL5 |

**Table 7: All the immune system genes common across the experimental group in Labrador**

**Supplementary table 12: Functional classification of Up-regulated genes of Ctrl vs Poly IC *Gaddi* dog according to the pathways they influence and control**

| **S. No.** | **Term** | **Count** | **Genes** |
| --- | --- | --- | --- |
| 1 | R-CFA-1280218~Adaptive Immune System | 5 | SNAP23, LCP2, ICOS, RNF111, LRRC41 |
| 2 | R-CFA-199991~Membrane Trafficking | 4 | STX16, SNAP23, TBC1D15, NAPG |
| 3 | R-CFA-5653656~Vesicle-mediated transport | 4 | STX16, SNAP23, TBC1D15, NAPG |
| 4 | cfa04660: T cell receptor signaling pathway | 4 | NFATC2, PPP2R5A, LCP2, ICOS |
| 5 | cfa04148: Efferocytosis | 4 | RXRA, TGFBRAP1, ATP2A2, NFATC2 |
| 6 | cfa05410: Hypertrophic cardiomyopathy | 3 | ATP2A2, ITGB8, PRKAG2 |
| 7 | R-CFA-6811440~Retrograde transport at the Trans-Golgi-Network | 2 | STX16, NAPG |
| 8 | R-CFA-6811438~Intra-Golgi traffic | 2 | STX16, NAPG |

**Supplementary table 13:**  **Functional classification of Down-regulated genes of Ctrl vs Poly IC *Gaddi* dog according to the pathways they influence and control**

| **S. No.** | **Term** | **Count** | **Genes** |
| --- | --- | --- | --- |
| 1 | cfa05200: Pathways in cancer | 8 | NOTCH2, IFNGR2, NCOA4, LEF1, MDM2, E2F3, JAK2, ETS1 |
| 2 | R-CFA-8953854~Metabolism of RNA | 7 | HNRNPA3, XPO1, PRPF18, RPL21, HNRNPR, SRSF5, EIF4B |
| 3 | cfa05166: Human T-cell leukemia virus 1 infection | 5 | FDPS, DLG1, XPO1, E2F3, ETS1 |
| 4 | cfa04919: Thyroid hormone signaling pathway | 4 | NOTCH2, NCOA2, MDM2, ATP1A1 |
| 5 | cfa05164: Influenza A | 4 | FDPS, XPO1, IFNGR2, JAK2 |
| 6 | cfa05169: Epstein-Barr virus infection | 4 | PSMD4, MDM2, TNFAIP3, E2F3 |
| 7 | R-CFA-72163~mRNA Splicing - Major Pathway | 4 | HNRNPA3, PRPF18, HNRNPR, SRSF5 |
| 8 | cfa04658: Th1 and Th2 cell differentiation | 3 | NOTCH2, IFNGR2, JAK2 |
| 9 | cfa03083: Polycom repressive complex | 3 | UBE2D2, LCOR, PHF19 |
| 10 | cfa01522: Endocrine resistance | 3 | NOTCH2, MDM2, E2F3 |
| 11 | cfa05215: Prostate cancer | 3 | LEF1, MDM2, E2F3 |
| 12 | R-CFA-156827~L13a-mediated translational silencing of Ceruloplasmin expression | 3 | EIF3M, RPL21, EIF4B |
| 13 | R-CFA-72706~GTP hydrolysis and joining of the 60S ribosomal subunit | 3 | EIF3M, RPL21, EIF4B |
| 14 | R-CFA-2559585~Oncogene Induced Senescence | 2 | MDM2, ETS1 |

**Supplementary table 14: Functional classification of Up-regulated genes of Ctrl vs LPS *Gaddi* dog according to the pathways they influence and control**

| **S. No.** | **Term** | **Count** | **Genes** |
| --- | --- | --- | --- |
| 1 | cfa05200: Pathways in cancer | 8 | NOTCH2, IFNGR2, NCOA4, LEF1, MDM2, E2F3, JAK2, ETS1 |
| 2 | R-CFA-8953854~Metabolism of RNA | 7 | HNRNPA3, XPO1, PRPF18, RPL21, HNRNPR, SRSF5, EIF4B |
| 3 | cfa05166: Human T-cell leukemia virus 1 infection | 5 | FDPS, DLG1, XPO1, E2F3, ETS1 |
| 4 | cfa04919: Thyroid hormone signaling pathway | 4 | NOTCH2, NCOA2, MDM2, ATP1A1 |
| 5 | cfa05164: Influenza A | 4 | FDPS, XPO1, IFNGR2, JAK2 |
| 6 | cfa05169: Epstein-Barr virus infection | 4 | PSMD4, MDM2, TNFAIP3, E2F3 |
| 7 | R-CFA-72163~mRNA Splicing - Major Pathway | 4 | HNRNPA3, PRPF18, HNRNPR, SRSF5 |
| 8 | cfa04658: Th1 and Th2 cell differentiation | 3 | NOTCH2, IFNGR2, JAK2 |
| 9 | cfa03083: Polycom repressive complex | 3 | UBE2D2, LCOR, PHF19 |
| 10 | cfa01522: Endocrine resistance | 3 | NOTCH2, MDM2, E2F3 |
| 11 | cfa05215: Prostate cancer | 3 | LEF1, MDM2, E2F3 |
| 12 | R-CFA-156827~L13a-mediated translational silencing of Ceruloplasmin expression | 3 | EIF3M, RPL21, EIF4B |
| 13 | R-CFA-72706~GTP hydrolysis and joining of the 60S ribosomal subunit | 3 | EIF3M, RPL21, EIF4B |
| 14 | R-CFA-2559585~Oncogene Induced Senescence | 2 | MDM2, ETS1 |
| 15 | R-CFA-168256~Immune System | 15 | CAP1, CD274, STAT3, SNAP23, UBR2, FOXO3, MMP8, HECTD3, ASB8, CXCR2, PTK2B, NLRP3, LCP2, SOCS5, LTF |
| 16 | R-CFA-9012999~RHO GTPase cycle | 7 | SNAP23, NCK2, PTK2B, MTR, ARHGAP5, OSBPL11, PICALM |
| 17 | R-CFA-194315~Signaling by Rho GTPases | 7 | SNAP23, NCK2, PTK2B, MTR, ARHGAP5, OSBPL11, PICALM |
| 18 | R-CFA-9716542~Signaling by Rho GTPases, Miro GTPases and RHOBTB3 | 7 | SNAP23, NCK2, PTK2B, MTR, ARHGAP5, OSBPL11, PICALM |
| 19 | R-CFA-1280218~Adaptive Immune System | 6 | CD274, HECTD3, ASB8, SNAP23, UBR2, LCP2 |
| 20 | R-CFA-6798695~Neutrophil degranulation | 5 | CAP1, SNAP23, CXCR2, MMP8, LTF |
| 21 | R-CFA-6785807~Interleukin-4 and Interleukin-13 signaling | 2 | STAT3, SOCS5 |

**Supplementary table 15:**  **Functional classification of Down-regulated genes of Ctrl vs LPS *Gaddi* dog according to the pathways they influence and control**

| **S. No.** | **Term** | **Count** | **Genes** |
| --- | --- | --- | --- |
| 1 | R-CFA-168256~Immune System | 12 | PYCARD, RAP2C, PSMA6, KLRB1, ADGRE5, CLTC, TNFAIP3, SLAMF7, RELA, NFKB1, SOCS5, SKP1 |
| 2 | R-CFA-73857~RNA Polymerase II Transcription | 9 | PSMA6, CNOT6, ELF1, TAF11, PAF1, POLR2G, MDM4, GTF2H3, SKP1 |
| 3 | R-CFA-74160~Gene expression (Transcription) | 9 | PSMA6, CNOT6, ELF1, TAF11, PAF1, POLR2G, MDM4, GTF2H3, SKP1 |
| 4 | R-CFA-212436~Generic Transcription Pathway | 8 | PSMA6, CNOT6, ELF1, TAF11, POLR2G, MDM4, GTF2H3, SKP1 |
| 5 | R-CFA-168249~Innate Immune System | 8 | PYCARD, RAP2C, PSMA6, ADGRE5, TNFAIP3, RELA, NFKB1, SKP1 |
| 6 | R-CFA-1280218~Adaptive Immune System | 7 | PSMA6, KLRB1, CLTC, SLAMF7, RELA, NFKB1, SKP1 |
| 7 | R-CFA-5607764~CLEC7A (Dectin-1) signaling | 5 | PYCARD, PSMA6, RELA, NFKB1, SKP1 |
| 8 | R-CFA-5621481~C-type lectin receptors (CLRs) | 5 | PYCARD, PSMA6, RELA, NFKB1, SKP1 |
| 9 | R-CFA-3700989~Transcriptional Regulation by TP53 | 5 | CNOT6, TAF11, POLR2G, MDM4, GTF2H3 |
| 10 | R-CFA-449147~Signaling by Interleukins | 5 | PSMA6, RELA, NFKB1, SOCS5, SKP1 |
| 11 | R-CFA-674695~RNA Polymerase II Pre-transcription Events | 4 | TAF11, PAF1, POLR2G, GTF2H3 |

**Supplementary table 16 : Functional classification of Up-regulated genes of Ctrl vs CpG *Gaddi* dog according to the pathways they influence and control**

| **S. No.** | **Term** | **Count** | **Genes** |
| --- | --- | --- | --- |
| 1 | cfa04151: PI3K-Akt signaling pathway | 17 | CSF3, LAMB3, CSF1, PPP2R5A, FASLG, PIK3R1, FOXO3, EREG, IKBKB, GYS1, GNG5, PPP2R2D, MLST8, ITGB8, IL7R, SGK1, BCL2L1 |
| 2 | cfa04621: NOD-like receptor signaling pathway | 14 | GSDMD, GABARAPL2, RIPK2, XIAP, MAPK14, IKBKB, PSTPIP1, MAPK8, STING1, OAS3, LOC488622, CAMP, NFKBIB, BCL2L1 |
| 3 | cfa05166: Human T-cell leukemia virus 1 infection | 14 | EGR1, TBP, IL1R2, ITGB2, XIAP, NFATC2, PIK3R1, IKBKB, MAPK8, KAT5, E2F1, EP300, PRKACB, BCL2L1 |
| 4 | cfa04010: MAPK signaling pathway | 14 | DUSP5, DUSP2, CSF1, FASLG, MAPK14, GADD45G, EREG, IKBKB, RAP1B, MAPK8, TAOK3, RASA1, RAPGEF2, PRKACB |
| 5 | cfa04062: Chemokine signaling pathway | 13 | CCL22, PIK3R1, FOXO3, IKBKB, RAP1B, HCK, GRK2, GNG5, CXCR2, CCR5, PRKACB, LOC480600, NFKBIB |
| 6 | cfa04014: Ras signaling pathway | 13 | RAB5C, CSF1, FASLG, PIK3R1, IKBKB, RAP1B, MAPK8, GNG5, RASA1, RGL1, PRKACB, RALGDS, BCL2L1 |
| 7 | cfa05132: Salmonella infection | 13 | GSDMD, CYFIP1, RAB5C, DCTN2, RIPK2, EXOC7, MAPK14, DNM2, IKBKB, CYTH2, DYNC1LI2, MAPK8, NCKAP1L |
| 8 | cfa05163: Human cytomegalovirus infection | 12 | IKBKB, STING1, GNG5, CXCR2, E2F1, NFATC2, FASLG, PIK3R1, CCR5, MAPK14, PRKACB, LOC480600 |
| 9 | cfa04060: Cytokine-cytokine receptor interaction | 12 | CSF3, CCL22, IL18RAP, TNFSF14, CSF1, IL1R2, CXCR2, FASLG, INHBA, CCR5, IL7R, LOC480600 |
| 10 | cfa04722: Neurotrophin signaling pathway | 11 | IKBKB, RAP1B, MAPK8, RIPK2, SORT1, RAPGEF1, FASLG, PIK3R1, FOXO3, MAPK14, NFKBIB |
| 11 | cfa04068: FoxO signaling pathway | 11 | IKBKB, GABARAPL2, MAPK8, EP300, FASLG, PIK3R1, FOXO3, MAPK14, SGK1, IL7R, GADD45G |
| 12 | cfa04210: Apoptosis | 11 | IKBKB, MAPK8, BCL2A1, PRF1, GZMB, XIAP, FASLG, PIK3R1, LMNB1, GADD45G, BCL2L1 |
| 13 | cfa04810: Regulation of actin cytoskeleton | 11 | CYFIP1, PIKFYVE, RGCC, ACTN1, RDX, ITGB2, ITGB8, NCKAP1L, PIK3R1, WASF2, MYH10 |
| 14 | cfa04380: Osteoclast differentiation | 10 | IKBKB, MAPK8, CSF1, FHL2, ACP5, NFATC2, LCP2, PIK3R1, MAPK14, FOSL2 |
| 15 | cfa04140: Autophagy - animal | 10 | GABARAPL2, RUBCN, MAPK8, MTMR3, TP53INP2, MLST8, WDFY3, PIK3R1, PRKACB, BCL2L1 |
| 16 | cfa05167: Kaposi sarcoma-associated herpesvirus infection | 10 | IKBKB, HCK, MAPK8, GNG5, E2F1, EP300, NFATC2, PIK3R1, CCR5, MAPK14 |
| 17 | cfa05170: Human immunodeficiency virus 1 infection | 10 | IKBKB, MAPK8, STING1, GNG5, NFATC2, FASLG, PIK3R1, CCR5, MAPK14, BCL2L1 |

**Supplementary table 17:** **Functional classification of Down-regulated genes of Ctrl vs CpG *Gaddi* dog according to the pathways they influence and control**

| **S. No.** | **Term** | **Count** | **Genes** |
| --- | --- | --- | --- |
| 1 | cfa05200: Pathways in cancer | 26 | ITGB1, NOTCH2, HSP90AB1, PLD1, ETS1, MAPK9, CASP8, TFG, CASP3, TPR, STAT4, IL12B, E2F3, JAK2, SKP1, STAT2, IFNGR2, NCOA4, TGFBR1, NFKB1, PLCB3, PIK3CA, MSH2, GNAS, IL7R, BCL2L1 |
| 2 | cfa05168: Herpes simplex virus 1 infection | 19 | DLA-DOB, DLA-DOA, STAT2, IFNGR2, LOC482938, IRAK4, ZNF23, LOC102152536, NFKB1, CASP8, PIK3CA, CASP3, ZNF746, IL12B, TNFRSF14, SRSF5, JAK2, ZNF354A, BCL2L1 |
| 3 | cfa05166: Human T-cell leukemia virus 1 infection | 17 | ATF2, DLA-DOB, CRTC3, DLA-DOA, ANAPC7, IL1R2, ITGB2, ITGAL, ETS1, NFKB1, TGFBR1, MAPK9, DLG1, CDC23, PIK3CA, E2F3, BCL2L1 |
| 4 | cfa05169: Epstein-Barr virus infection | 16 | DLA-DOB, PSMD12, DLA-DOA, STAT2, IRAK4, ITGAL, NFKB1, CXCL10, MAPK9, PSMC6, CASP8, PIK3CA, PSMD4, CASP3, E2F3, CD44 |
| 5 | cfa05132: Salmonella infection | 16 | HSP90AB1, IRAK4, PIK3C2A, NFKB1, PYCARD, MAPK9, PTPRC, LOC106557476, CASP8, PIK3CA, CASP3, RAB9A, FLNA, KPNA3, VPS39, SKP1 |
| 6 | cfa05165: Human papillomavirus infection | 16 | ITGB1, NOTCH2, MX2, STAT2, ITGA1, PSEN1, NFKB1, DLG1, PKM, CASP8, PIK3CA, CASP3, TADA3, SPP1, GNAS, ATP6V1C1 |
| 7 | cfa05164: Influenza A | 14 | DLA-DOB, DLA-DOA, MX2, STAT2, IFNGR2, IRAK4, NFKB1, PYCARD, CXCL10, CASP8, PIK3CA, CASP3, IL12B, JAK2 |
| 8 | cfa05417: Lipid and atherosclerosis | 14 | HSP90AB1, IRAK4, NFKB1, PYCARD, MAPK9, PLCB3, CASP8, RAP1A, PIK3CA, CASP3, IL12B, CD36, JAK2, BCL2L1 |
| 9 | cfa04010: MAPK signaling pathway | 14 | MAP3K2, ATF2, MEF2C, PLA2G4A, CACNA1A, IRAK4, NFKB1, TGFBR1, MAPK9, RAP1A, CASP3, FLNA, PAK2, MAP3K12 |
| 10 | cfa05152: Tuberculosis | 13 | DLA-DOB, DLA-DOA, IFNGR2, ITGB2, IRAK4, NFKB1, MAPK9, CASP8, CASP3, LAMP2, MRC1, IL12B, JAK2 |
| 11 | cfa05145: Toxoplasmosis | 12 | ITGB1, MAPK9, DLA-DOB, DLA-DOA, CASP8, CASP3, IFNGR2, IL12B, IRAK4, JAK2, NFKB1, BCL2L1 |
| 12 | cfa05162: Measles | 12 | MAPK9, CASP8, PIK3CA, STAT2, MX2, CASP3, MSN, RAB9A, IL12B, IRAK4, NFKB1, BCL2L1 |
| 13 | cfa05161: Hepatitis B | 12 | ATF2, MAPK9, CASP8, PIK3CA, STAT2, CASP3, STAT4, E2F3, IRAK4, JAK2, TGFBR1, NFKB1 |
| 14 | cfa04620: Toll-like receptor signaling pathway | 11 | CXCL10, MAPK9, CASP8, PIK3CA, STAT2, CD80, SPP1, IL12B, IRF5, IRAK4, NFKB1 |
| 15 | cfa05142: Chagas disease | 11 | MAPK9, PLCB3, CASP8, PIK3CA, IFNGR2, GNAS, IL12B, CFLAR, IRAK4, TGFBR1, NFKB1 |
| 16 | cfa04621: NOD-like receptor signaling pathway | 11 | PYCARD, P2RX7, MAPK9, PLCB3, HSP90AB1, CASP8, STAT2, ERBIN, IRAK4, NFKB1, BCL2L1 |
| 17 | cfa04120: Ubiquitin mediated proteolysis | 11 | DET1, ITCH, COP1, CDC23, CUL5, ANAPC7, SYVN1, UBE2E1, TRIP12, PIAS2, SKP1 |
| 18 | cfa04668: TNF signaling pathway | 10 | ATF2, CXCL10, MAPK9, ITCH, BAG4, CASP8, PIK3CA, CASP3, CFLAR, NFKB1 |
| 19 | cfa04514: Cell adhesion molecules | 10 | ITGB1, DLA-DOB, SELPLG, ALCAM, DLA-DOA, PTPRC, SELL, CD80, ITGB2, ITGAL |
| 20 | cfa04145: Phagosome | 10 | ITGB1, SCARB1, DLA-DOB, DLA-DOA, LOC106557476, LAMP2, ITGB2, MRC1, CD36, ATP6V1C1 |
| 21 | cfa05160: Hepatitis C | 10 | SCARB1, CXCL10, CASP8, PIK3CA, STAT2, MX2, CASP3, E2F3, CFLAR, NFKB1 |
| 22 | cfa04630: JAK-STAT signaling pathway | 10 | PIK3CA, STAT2, IFNGR2, IL24, STAT4, IL12B, JAK2, IL7R, PIAS2, BCL2L1 |
| 23 | cfa04110: Cell cycle | 10 | MAU2, STAG1, NIPBL, CDC23, DBF4, ESCO1, ANAPC7, ORC3, E2F3, SKP1 |
